# A Genome-Wide Screen Identifies Genes in Rhizosphere-Associated *Pseudomonas* Required to Evade Plant Defenses

**DOI:** 10.1128/mBio.00433-18

**Published:** 2018-11-06

**Authors:** Zhexian Liu, Polina Beskrovnaya, Ryan A. Melnyk, Sarzana S. Hossain, Sophie Khorasani, Lucy R. O’Sullivan, Christina L. Wiesmann, Jen Bush, Joël D. Richard, Cara H. Haney

**Affiliations:** a Department of Microbiology and Immunology, The University of British Columbia, Vancouver, Canada; b Department of Molecular Biology, Massachusetts General Hospital, Boston, Massachusetts, USA; c Michael Smith Laboratories, The University of British Columbia, Vancouver, Canada; University of California, Berkeley

**Keywords:** *Arabidopsis*, *Pseudomonas*, pattern-triggered immunity, phosphodiesterase, putrescine, rhizosphere

## Abstract

Pseudomonas fluorescens and related plant root (“rhizosphere”)-associated species contribute to plant health by modulating defenses and facilitating nutrient uptake. To identify bacterial fitness determinants in the rhizosphere of the model plant Arabidopsis thaliana, we performed a high-throughput transposon sequencing (Tn-Seq) screen using the biocontrol and growth-promoting strain *Pseudomonas* sp. WCS365. The screen, which was performed in parallel on wild-type and immunocompromised *Arabidopsis* plants, identified 231 genes that increased fitness in the rhizosphere of wild-type plants. A subset of these genes decreased fitness in the rhizosphere of immunocompromised plants. We hypothesized that these genes might be involved in avoiding plant defenses and verified 7 *Pseudomonas* sp. WCS365 candidate genes by generating clean deletions. We found that two of these deletion mutants, Δ*morA* (encoding a putative diguanylate cyclase/phosphodiesterase) and Δ*spuC* (encoding a putrescine aminotransferase), formed enhanced biofilms and inhibited plant growth. We found that mutants *ΔspuC* and *ΔmorA* induced pattern-triggered immunity (PTI) as measured by induction of an *Arabidopsis* PTI reporter and *FLS2*/*BAK1-*dependent inhibition of plant growth. We show that MorA acts as a phosphodiesterase to inhibit biofilm formation, suggesting a possible role in biofilm dispersal. We found that both putrescine and its precursor arginine promote biofilm formation that is enhanced in the *ΔspuC* mutant, which cannot break down putrescine, suggesting that putrescine might serve as a signaling molecule in the rhizosphere. Collectively, this work identified novel bacterial factors required to evade plant defenses in the rhizosphere.

## Abstract (precis)

While rhizosphere bacteria hold the potential to improve plant health and fitness, little is known about the bacterial genes required to evade host immunity. Using a model system consisting of *Arabidopsis* and a beneficial *Pseudomonas* sp. isolate, we identified bacterial genes required for both rhizosphere fitness and for evading host immune responses. This work advances our understanding of how evasion of host defenses contributes to survival in the rhizosphere.

## INTRODUCTION

Plant root-associated commensal microbes confer fitness advantages to plant hosts, including growth promotion, nutrient uptake, and resistance to pathogens (1). In order to benefit its plant host, a root-associated microbe must survive in the rhizosphere, compete for plant-derived nutrients, and avoid plant defenses. Despite the importance of rhizosphere competence for microbes to confer benefits to plants, the mechanisms regulating rhizosphere fitness and evasion of host defenses are poorly understood.

Symbiotic bacteria must cope with a host immune system, which can recognize microbe-associated molecular patterns (MAMPs) such as flagellin, lipopolysaccharide, and chitin and trigger a defense response. Plant-associated Pseudomonas fluorescens and related species can suppress local plant defenses (2) and trigger expression of MAMP-inducible genes (3). Using forward genetics approaches, genes required for rhizosphere competence have been identified in *Pseudomonas* spp. (4–7), including genes required for motility and nutrient uptake (4, 5). However, these factors have not been linked to evasion of plant immunity. How beneficial bacteria navigate host immune surveillance and manage to survive despite host defense responses remains elusive.

To identify fitness determinants in the presence of a plant immune system, we performed transposon mutagenesis coupled with high-throughput transposon sequencing (Tn-Seq) using *Pseudomonas* sp. WCS365 on wild-type and immunocompromised *Arabidopsis* (see below). *Pseudomonas* sp. WCS365 is a growth promotion and biocontrol strain (6, 8, 9) and has been studied as a model for biofilm formation *in vitro* (10) and for genes important in rhizosphere colonization (6). Tn-Seq is a high-throughput technique to rapidly assess fitness of each gene in a bacterial genome in a single experiment (11) and has been shown to be a particularly powerful method to identify determinants of bacterial fitness in association with animals (12–14) and plants (4). We reasoned that Tn-Seq might be an efficient method to rapidly identify bacterial genes required to avoid or suppress host immunity in the rhizosphere.

Here we report a Tn-Seq screen that identified 231 genes required for *Pseudomonas* sp. WCS365 fitness in the rhizosphere of wild-type *Arabidopsis* plants. We followed up on a subset of candidate genes that increase fitness in the wild-type Col-0 rhizosphere but that decrease fitness in the rhizosphere of a quadruple immune hormone mutant containing mutations in *DDE2*, *EIN2*, *PAD4*, and *SID2* (*deps* [15]). We found that two mutants, *ΔspuC* and *ΔmorA*, induced pattern-triggered immunity (PTI) in *Arabidopsis* via the flagellin receptor *FLS2*. We provide evidence that *Pseudomonas* sp. WCS365 *morA* and *spuC* temper biofilm formation and are required for evasion of plant immunity.

## RESULTS

### A Tn-Seq screen identified *Pseudomonas* sp. WCS365 fitness determinants in the *Arabidopsis* rhizosphere.

To identify genes required for *Pseudomonas* sp. WCS365 fitness in the *Arabidopsis* rhizosphere, we performed a large-scale *mariner* transposon mutagenesis screen followed by next-generation sequencing (Tn-Seq) (see Materials and Methods for details). For this screen, germfree *Arabidopsis* plants were grown in a sterile calcine clay and perlite mix with a plant nutrient solution (no carbon) to support plant growth. The screen was performed in parallel on wild-type *Arabidopsis* Col-0 and a mutant impaired in multiple immune hormone signaling pathways (*dde2-1*, *ein2-1*, *pad4-1*, and *sid2-2*; *“deps”* mutant) (15). We chose the immunocompromised *deps* mutant because it exhibited 5-fold to 10-fold-higher growth of *Pseudomonas* sp. WCS365 in the rhizosphere than wild-type plants (see Fig. S1A in the supplemental material). No-plant controls were supplemented with 20 mM succinate to support bacterial growth (Fig. S1B and C). We reasoned that insertions in WCS365 genes required for evasion of plant immunity would result in decreased fitness on wild-type plants but not on the immunocompromised *deps* mutant, allowing us to distinguish general colonization determinants from genes required for avoidance or suppression of plant immunity.

10.1128/mBio.00433-18.1 FIG S1 Treatments and setup for the Tn-Seq experiment. (A) Wild-type and mutant *Arabidopsis* plants were grown in a sterile clay mix and inoculated with 10^4^ CFU *Pseudomonas* sp. WCS365/plant. Root CFU/gram levels were measured 1 week later by homogenizing plant roots and plating to count CFUs. Levels designate significance by ANOVA and Tukey’s HSD. (B) Plants were grown in a sterile soil-like mix of calcine clay, sand, and pearlite (1:1:1) saturated with 0.5× MS media with no carbon source and inoculated with a transposon insertion library of *Pseudomonas* sp. WCS365. The no-plant control was inoculated with 20 mM succinate to allow bacterial growth. Soil cores or plant roots were harvested 1 week after inoculation. Cores or roots with attached soil were used for DNA isolation. (C) Final growth of bacteria in soil mix with no carbon or in soil mix with succinate or in the rhizosphere of Col-0 or the quadruple hormone mutant. Download FIG S1, PDF file, 0.2 MB. Copyright © 2018 Liu et al. 2018 Liu et al. This content is distributed under the terms of the Creative Commons Attribution 4.0 International license.

We sequenced the genome of *Pseudomonas* sp. WCS365 (see Materials and Methods) to facilitate identification of transposon insertion sites in our Tn-Seq library (GenBank accession no. PHHS01000000). To determine placement within the genus *Pseudomonas*, we generated a phylogenomic tree using 381 housekeeping genes identified by PhyloPhlAn (16) (Fig. S2). We found that *Pseudomonas* sp. WCS365 falls within the P. fluorescens group of the fluorescent pseudomonads and is a close relative of *Pseudomonas* sp. NFM421 within the P. brassicacearum subgroup (17).

10.1128/mBio.00433-18.2 FIG S2 Phylogenomic analysis places *Pseudomonas* sp. WCS365 within the P. brassicacearum subgroup of the P. fluorescens group. The genome of *Pseudomonas* sp. WCS365 was sequenced, and a phylogenomic tree containing other *Pseudomonas* spp. was generated using PhyloPhlAn (16). Download FIG S2, PDF file, 0.2 MB. Copyright © 2018 Liu et al. 2018 Liu et al. This content is distributed under the terms of the Creative Commons Attribution 4.0 International license.

**Fig 1:**
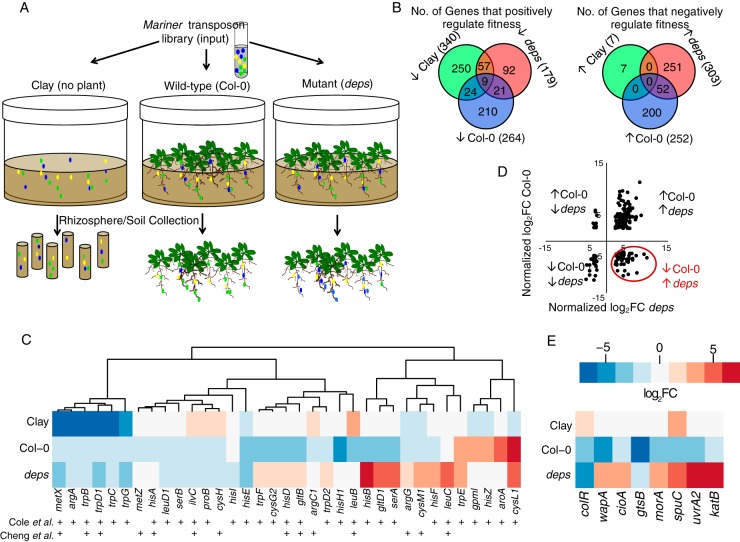
A Tn-Seq screen identified *Pseudomonas* sp. WCS365 genes that affect fitness in the rhizospheres of wild-type and immunocompromised *Arabidopsis*. (A) A transposon insertion library was added to sterile clay with no plants and 20 mM succinate, the roots of wild-type Col-0 plants, or an immunocompromised mutant (*deps*). (B) The Tn-Seq screen identified genes that increase or decrease fitness in the rhizosphere of wild-type Col-0 plants or an immunocompromised mutant. (C) Amino acid biosynthetic genes previously shown to have positive effects on fitness (Cole et al. [4]) or negative effects on growth promotion (Cheng et al. [5]) in rhizosphere-associated *Pseudomonas* spp. (D) We focused on genes with a fitness cost in the immunocompromised *deps* rhizosphere but that provided a fitness advantage in the Col-0 rhizosphere. (E) Candidate genes chosen for follow-up showed significant differences with respect to fitness in the Col-0 versus *deps* rhizospheres (*colR* was chosen as a control).

To identify bacterial genes required for WCS365 fitness in the *Arabidopsis* rhizosphere, 3-week-old plants were inoculated with a Tn-Seq library containing insertions in 66,894 TA dinucleotide sites distributed across the genome with approximately 9.8 insertions per 1,000 bp (Materials and Methods) (see also Fig. S3 and Data Set S1 in the supplemental material). Plants were inoculated with 10^4^ CFU per plant, and plant roots or no-plant controls were harvested 1 week later (Fig. 1A; see also Fig. S1B). We sequenced the transposon junctions in the rhizosphere and soil samples and compared the relative abundances of insertions in the rhizosphere of Col-0, the immunocompromised *deps* mutant, or the no-plant control relative to the input. In our screen, we observed a significant bottleneck and ∼35% of insertions were lost under any given treatment condition. Bottlenecks have previously been observed for other host-associated Tn-Seq screens (14). We adjusted our analysis to account for bottlenecks by first combining all reads per gene and then averaging the 3 replicates per gene (18).

10.1128/mBio.00433-18.3 FIG S3 Library construction and frequency of insertions by genes in the Tn-Seq input library. (A) Primers are shown in Data Set S1 in the supplemental material. (Step 1) After DNA isolation, DNA was digested with MmeI to cleave 21 bp upstream and downstream of the transposon insertion. (Step 2) Digested DNA was subjected to phosphatase treatment. (Step 3) Double-stranded adapters were ligated onto DNA that had been subjected to phosphatase treatment. (Step 4) The region flanking the transposon junctions was PCR amplified using transposon-specific (PCR.X) and adapter-specific (U.) primers. (B) The input library was sequenced and mapped to the WCS365 genome. We found a mean of 10.3 insertions and a median of 8 insertions per gene. Download FIG S3, PDF file, 1.0 MB. Copyright © 2018 Liu et al. 2018 Liu et al. This content is distributed under the terms of the Creative Commons Attribution 4.0 International license.

10.1128/mBio.00433-18.10 DATA SET S1 *Pseudomonas* sp. WCS365 Tn-Seq fitness data and primer sequences. Tabs 1 to 7 show fitness data for all sampled insertions in the rhizosphere of wild-type Col-0 plants, the *deps* mutant, or no plant controls. Tabs 8 and 9 show fitness data for putrescine genes, and orthologues of WCS365 genes. Tabs 10 and 11 show primer sequences for the Tn-Seq experiments and construction of deletion strains. Download Data Set S1, XLSX file, 0.9 MB. Copyright © 2018 Liu et al. 2018 Liu et al. This content is distributed under the terms of the Creative Commons Attribution 4.0 International license.

We identified 231 genes that significantly increased fitness in the wild-type Col-0 rhizosphere (insertions in these genes caused a decrease in relative fitness) (Fig. 1B; see also Data Set S1). We found an additional 113 genes that increased fitness in the immunocompromised *deps* rhizosphere but only 21 genes that increased fitness in the rhizosphere of both plants. We also found genes that decreased fitness in the rhizosphere of the wild-type strain and the immunocompromised *deps* mutant (insertions in these genes increased relative fitness in the rhizosphere), including 52 genes that decreased fitness on both plant genotypes.

We compared the genes identified in our Tn-Seq screen to those identified in several recent screens for genes that affect the fitness or growth promotion ability of rhizosphere-associated *Pseudomonas* spp. (4, 5). Cole et al. (2017) found that insertions in amino acid biosynthesis genes resulted in a fitness advantage of *Pseudomonas* sp. WCS417 in the rhizosphere (4), while Cheng et al. (2017) found that insertions in orthologs of the WCS417 amino acid biosynthesis genes rendered *Pseudomonas* sp. SS101 unable to promote plant growth or provide protection from pathogens (5). We specifically looked at this same set of amino acid biosynthesis genes in our data set (see Data Set S1 in the supplemental material) and found that the majority of insertions in *Pseudomonas* sp. WCS365 amino acid biosynthesis genes reduced rhizosphere fitness in our study (Fig. 1C). Of note, a significant portion of insertions in these genes increased rhizosphere fitness in the immunocompromised *deps* background (Fig. 1C). These results indicate that an inability to synthesize certain amino acids resulted in a fitness defect in the wild-type Col-0 rhizosphere under the conditions used in our study. These data also suggest that there may be altered amino acid profiles between the rhizosphere of wild-type plants and that of the immunocompromised *deps* mutant.

### Confirmation of the role in rhizosphere fitness of genes identified by Tn-Seq.

We hypothesized that bacterial genes that provide a fitness advantage in the presence of plant defenses might confer a fitness disadvantage in the absence of defense responses. As a result, we considered genes that had a large negative log_2_ fold change ratio for fitness on wild-type Col-0 plants versus the immunocompromised *deps* mutant. We found that a significant portion of the genes that increased fitness in the Col-0 rhizosphere had negative effects on bacterial fitness in the immunocompromised *deps* rhizosphere (Fig. 1D and E; see also Data Set S1). To determine if we had successfully identified genes involved in the survival of plant defenses, we independently tested 7 *Pseudomonas* sp. WCS365 candidates that increased fitness in the Col-0 rhizosphere but decreased fitness in the immunocompromised *deps* rhizosphere (Fig. 1E) (Table 1). We cleanly deleted genes encoding a catalase (*katB*), a diguanylate cyclase (DGC)/phosphodiesterase (PDE) (*morA*), a putrescine aminotransferase (*spuC*), an excinuclease (*uvrA*), a cytochrome oxidase subunit (*cioA*), an ABC transporter permease (*gtsB*), and a putative secreted protein (*wapA*) (Table 1; annotations were performed as described in Materials and Methods). The previously identified colonization factor *colR* (7) was found to have impaired fitness in the rhizosphere of both wild-type plants and the immunocompromised *deps* mutant (Fig. 1E) (Table 1) and was deleted as a control.

**TABLE 1:** Genes selected for further characterization include those with a large different in fitness in the rhizospheres of wild-type (Col-0) and immunocompromised (*deps*) rhizospheres^*a*^

Gene ID	Gene name	Predicted function	No. of insertion sites	Log_2_FC (clay)	*P* value	Log_2_FC (Col-0)	*P* value	Log_2_FC (*deps*)	*P* value	Log_2_FC (Col-0/ *deps*)
WCS365_05599	*wapA*	Nuclease	61	0.6	0.493	−2.9	0.040	3.1	0.003	−6.02
WCS365_04646	*cioA*	Cytochrome oxidase subunit	5	0.5	0.974	−1.8	0.012	2.5	0.001	−4.25
WCS365_04136	*gtsB*	Glucose ABC transporter permease	5	0.4	0.662	−6.6	0.090	−1.4	0.166	−5.18
WCS365_05664	*morA*	Cyclic di-GMP phosphodiesterase	48	0.7	0.169	−2.4	0.004	2.9	0.079	−5.29
WCS365_00305	*spuC*	Putrescine aminotransferase	13	3.0	0.071	−2.8	0.092	4.9	0.045	−7.68
WCS365_05132	*uvrA*	Excinuclease	28	0.6	0.431	−2.4	0.014	5.2	0.013	−7.60
WCS365_04639	*katB*	Catalase	18	0.2	0.701	−1.4	0.134	5.4	0.012	−6.75
WCS365_04320	*colR*	Response regulator	6	1.7	0.147	−2.9	0.002	−3.7	0.065	0.84

a Log_2_FC of the fitness of Col-0 relative to *deps* was calculated by first normalizing fitness data to the fitness in clay and then taking the ratio. ID, identifier.

**Fig 2:**
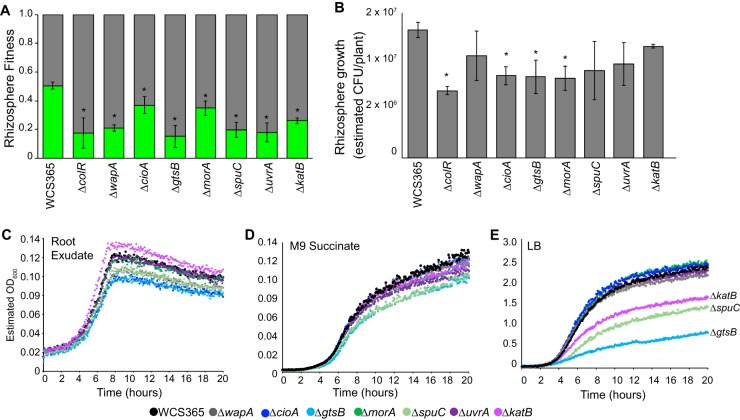
The Tn-Seq screen identified *Pseudomonas* sp. WCS365 genes required for rhizosphere fitness. (A and B) Clean deletions were generated in *Pseudomonas* sp. WCS365 candidate genes and tested for fitness (A) or growth (B) in the *Arabidopsis* rhizosphere. Plants were grown in 48-well plates in hydroponic media. For fitness assays (A), plants were cocolonized with a wild-type strain expressing mCherry and a mutant or wild-type strain expressing GFP. The relative levels of abundance were read after 3 days. For rhizosphere growth assays (B), plants were colonized with mutant or wild-type strains expressing GFP, and fluorescence was measured as a proxy for growth. *, *P < *0.01 (by analysis of variance [ANOVA] and Tukey’s honestly significant difference [HSD] test relative to wild-type *Pseudomonas* sp. WCS365). (C to E) Growth of mutants in competition with wild-type cells in (C) M9 plus root exudate or (D) M9 plus 30 mM succinate or (E) LB. Mutants expressing a GFP plasmid were grown in competition with the wild-type strain expressing mCherry, and growth of the GFP-expressing mutant was quantified with a plate reader (see Table S1 and S2).

We retested our 7 *Pseudomonas* sp. WCS365 deletion mutants for growth and fitness in the *Arabidopsis* rhizosphere using a previously described hydroponic assay (9). We cotreated plants with wild-type *Pseudomonas* sp. WCS365 expressing a red fluorescent protein (mCherry) from a plasmid and the *Pseudomonas* sp. WCS365 deletion strains expressing green fluorescent protein (GFP) from the same plasmid backbone. We then quantified relative fluorescence levels as a measure of fitness. Under these conditions, all 7 strains, along with the *ΔcolR* control, had significant rhizosphere fitness defects (Fig. 2A). We tested the strains individually for colonization and found that a subset had significant growth defects in the rhizosphere (Fig. 2B). Collectively, these results indicate that the Tn-Seq screen successfully identified novel *Pseudomonas* sp. WCS365 genes required for fitness in the *Arabidopsis* rhizosphere.

10.1128/mBio.00433-18.8 TABLE S1 Doubling times for *Pseudomonas* sp. WCS365 mutants *in planta* and *in vitro*. Mutants were grown in LB, M9 plus 30 mM succinate, or M9 with root exudate as the sole carbon source. Bacterial OD_600_ was measured with a plate reader (see Fig. S5), and doubling time was calculated based on growth during the exponential stage. Relative fitness was calculated as the fraction of the final culture that consisted of the designated mutant strain grown in competition with the wild-type strain. The rhizosphere data are shown in Fig. 2A, and the levels in LB, M9 succinate, and M9 root exudate were calculated based on 20 h of *in vitro* growth and are shown in Fig. 2C to E. *, *P < *0.05; **, *P < *0.01 relative to wild-type *Pseudomonas* sp. WCS365 by ANOVA and Tukey’s HSD. Download Table S1, DOCX file, 0.1 MB. Copyright © 2018 Liu et al. 2018 Liu et al. This content is distributed under the terms of the Creative Commons Attribution 4.0 International license.

10.1128/mBio.00433-18.9 TABLE S2 Fitness of *Pseudomonas* sp. WCS365 mutants *in planta* and *in vitro*. Relative fitness was calculated as the fraction of the final culture corresponding to the designated mutant strain grown in competition with the wild-type strain. The rhizosphere data are shown in Fig. 2A, and the levels in LB, M9 succinate, and M9 root exudate were calculated based on 20 h of *in vitro* growth; the results are shown in Fig. 2C to E. *, *P < *0.05; **, *P < *0.01 relative to wild-type *Pseudomonas* sp. WCS365 by ANOVA and Tukey’s HSD. Download Table S2, DOCX file, 0.1 MB. Copyright © 2018 Liu et al. 2018 Liu et al. This content is distributed under the terms of the Creative Commons Attribution 4.0 International license.

A previous screen for *Pseudomonas* fitness determinants in the rhizosphere identified mutants with poor or no growth in both minimal media and the rhizosphere (5). To determine if general growth defects underlie the observed rhizosphere fitness defects, we performed growth curves using root exudate as a sole carbon source and measured *in vitro* bacterial growth and fitness. We found that all of the mutants showed nearly normal growth rates (as measured by doubling time) when grown alone or in competition with the wild-type strain (GFP mutant and mCherry wild-type strain) in root exudate (Fig. 2C; see also Fig. S4A). We found that a subset of the mutants had significant fitness defects in LB (mutants *ΔspuC*, *ΔgtsB*, and *ΔkatB*) or minimal media (mutants *ΔspuC* and *ΔgtsB*) as quantified by the fraction of the final culture that was composed of the mutant strain (Fig. 2C to E; see also Fig. S4A to C and Table S1 and S2 in the supplemental material). Because all strains could grow with root exudate as a sole carbon source, these data suggest that the fitness defects were specific to the presence of a live plant or might have been related to non-carbon-related rhizosphere nutrients.

10.1128/mBio.00433-18.4 FIG S4 Growth of *Pseudomonas* sp. WCS365 mutants in rich and minimal media and the *ΔgtsB* mutant in dextrose. (A to C) Growth of *Pseudomonas* sp. WCS365 and mutants in (A) M9 plus root exudate, (B) M9 plus 30 mM succinate, and (C) LB. For all assays, *Pseudomonas* sp. WCS365 cells were grown overnight and then diluted to an estimated OD_600_ = 0.03. Bacteria were grown for 24 h in a shaking plate reader, with readings taken every 15 min. (D to F) The *ΔgtsB* mutant was grown in (A) LB, (B) M9 plus 30 mM succinate, or (C) M9 plus 20 mM dextrose. Download FIG S4, PDF file, 1.1 MB. Copyright © 2018 Liu et al. 2018 Liu et al. This content is distributed under the terms of the Creative Commons Attribution 4.0 International license.

To gain insights into the requirements of these 7 mutants in the plant rhizosphere, we surveyed a publicly available fitness database where barcoded transposon libraries were assessed for fitness under *in vitro* conditions (19). We identified the loci with the highest similarity to the WCS365 predicted protein sequences for the two most closely related strains, *Pseudomonas* sp. FW300-N2E2 and FW300-N2C3 (see Data Set S1 in the supplemental material). Insertions in N2E2 and N2C3 *morA*, *wapA*, and *katB* were fitness neutral under all conditions tested (Data Set S1). Insertions in *gtsB* resulted in pleotropic fitness defects, including growth with glucose and galactose as sole carbon sources. Insertions in *spuC* resulted in growth defects with putrescine used as a sole carbon or nitrogen source, supporting the idea of a potential role in putrescine metabolism. Insertions in *uvrA* resulted in fitness defects in the presence of DNA-damaging agents, supporting the idea of a role in DNA repair. Collectively, these data suggest that loss of these genes (with the exception of *gtsB*) does not result in pleotropic growth defects but results instead in defects that are specific to a limited number of conditions that may be relevant for rhizosphere growth.

While the majority of the 7 deletion mutants did not show growth or fitness defects *in vitro*, deletion of a predicted glucose transporter, *gtsB*, resulted in impaired growth rates and fitness of *Pseudomonas* sp. WCS365 in LB media (Fig. 2E; see also Table S1 and S2). In the event that the defect was due to a second site mutation, we reconstructed the *ΔgtsB* strain and independently confirmed the growth and fitness defect in LB. To test if *Pseudomonas* sp. WCS365 *gtsB* has a role in glucose transport, we tested the *ΔgtsB* mutant for growth in minimal media with succinate or dextrose as the sole carbon source. We found that the *ΔgtsB* mutant has a significant growth defect on dextrose but not succinate (Fig. S4D and E), consistent with its predicted role as a glucose transporter. Because glucose is not the dominant carbon source in LB media, it is unclear why this mutant would have a fitness defect in LB. As a result, it is unclear whether the *ΔgtsB* mutant fails in the rhizosphere due to an inability to transport glucose or due to pleotropic effects of deletion of this transporter component.

### *Pseudomonas* sp. WCS365 *ΔmorA* and *ΔspuC* mutants induce pattern-triggered immunity.

When applied to wild-type Arabidopsis thaliana Col-0, *Pseudomonas* sp. WCS365 promotes plant growth as measured by increased plant weight and increased density of lateral roots (9). Microbe-associated molecular patterns (MAMPs) can be sensed by plants, including *Arabidopsis*, via interaction with pattern recognition receptors (PRRs), resulting in defense responses that include callose deposition, inhibition of primary root growth, and induction of defense-related gene expression (collectively called “pattern-triggered immunity” [PTI]) (2, 3). We therefore hypothesized that if any of the *Pseudomonas* sp. WCS365 genes identified in our screen are required for evasion or suppression of immunity, the deletion mutants might trigger PTI as measured by plant growth inhibition and induction of defense-related genes.

**Fig 3:**
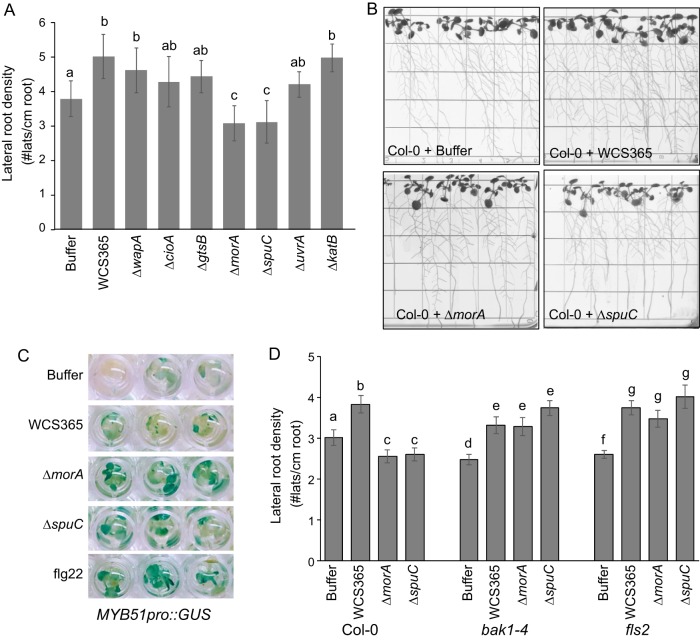
*Pseudomonas* sp. WCS365 *ΔmorA* and *ΔspuC* mutants induce pattern-triggered immunity. (A) The growth promotion ability of WCS365 mutants was tested on wild-type Arabidopsis thaliana ecotype Col-0. Lateral root density (number of lateral roots per centimeter of primary root [#lats/cm root]) is shown. (B) Images of growth promotion assays showing the *ΔmorA* and *ΔspuC* mutants. Results of PGP assays performed with the remainder of the *Pseudomonas* sp. WCS365 mutants are shown in Fig. S5. (C) Using an *Arabidopsis* MAMP-inducible transgenic reporter line (*MYB51pro*::*GUS*), we found that *Pseudomonas* sp. WCS365 *ΔmorA* and *ΔspuC* mutants induce MAMP-dependent gene expression. (D) *Arabidopsis* growth inhibition by the *ΔmorA* and *ΔspuC* mutants is dependent on MAMP perception via *BAK1* and *FLS2.* In panels A and D, letters designate levels of significance (*P *< 0.05) within a plant genotype as determined by ANOVA and Tukey’s HSD test.

Under conditions where wild-type WCS365 promotes plant growth, we found that two of the seven mutants, the *ΔmorA* and *ΔspuC* mutants, inhibited plant growth as measured by a reduction in plant lateral root density and primary root elongation (Fig. 3A and B; see also Fig. S5). The remaining mutants, including the *ΔgtsB* mutant, which had the most severe rhizosphere growth and fitness defect (Fig. 2A and B), still triggered an increase in *Arabidopsis* lateral root density. We tested an *Arabidopsis* reporter line consisting of the promoter of MAMP-inducible gene *MYB51* fused to the ß-glucuronidase (*MYB51pro*::*GUS*) reporter gene, which provides a qualitative readout of PTI (2). We found slight induction of *MYB51* by wild-type *Pseudomonas* sp. WCS365 and enhanced *MYB51* expression in seedlings exposed to *ΔmorA*, *ΔspuC*, or an epitope of bacterial flagellin that triggers a plant immune response, flg22 (Fig. 3C). Collectively, these data suggest that *morA* and *spuC* are required to avoid triggering PTI in *Arabidopsis*.

10.1128/mBio.00433-18.5 FIG S5 Images of *Pseudomonas* sp. WCS365 plant growth promotion (PGP) assays. Plants were grown on plates and inoculated with wild-type or mutant *Pseudomonas* sp. WCS365 with 3 µl bacteria at a final OD_600_ of 0.01. Plates were imaged 10 days later, and lateral root density was calculated; the results are shown in Fig. S3. Download FIG S5, PDF file, 0.6 MB. Copyright © 2018 Liu et al. 2018 Liu et al. This content is distributed under the terms of the Creative Commons Attribution 4.0 International license.

*Arabidopsis* perception of the majority of MAMPs, including flagellin, is dependent on the presence of the coreceptor BAK1 (20). We therefore tested if growth promotion by *ΔmorA* and *ΔspuC* is restored in a *bak1-4* mutant. We observed significant growth promotion of an *Arabidopsis bak1-4* mutant by *Pseudomonas* sp. WCS365 *ΔmorA* and *ΔspuC* (Fig. 3D). These data indicate that the *ΔmorA* and *ΔspuC* mutants inhibit plant growth due to induction of PTI via *BAK1*.

Motility is necessary for rhizosphere colonization by a number of microbes (21); however, failure to downregulate motility might trigger PTI, as *Arabidopsis* can sense flagellin produced by *Pseudomonas* spp. We tested if growth inhibition was dependent on the plant flagellin perception by testing an *Arabidopsis fls2* mutant that cannot sense bacterial flagellin (22). We observed significant growth promotion of an *Arabidopsis fls2* mutant by *Pseudomonas* sp. WCS365 *ΔmorA* and *ΔspuC* (Fig. 3D). These data indicate that *Arabidopsis* flagellin perception underlies the defense response triggered by *ΔmorA* and *ΔspuC* mutants.

### *Pseudomonas* sp. WCS365 *ΔmorA* and *ΔspuC* mutants form enhanced biofilms without defects in motility.

**Fig 4:**
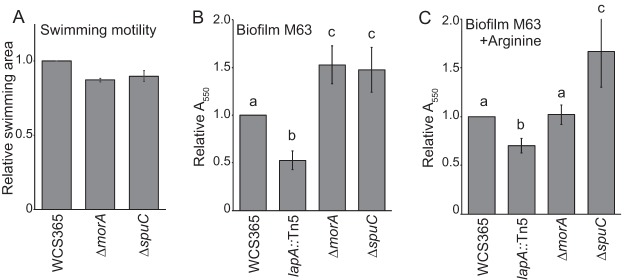
*Pseudomonas* sp. WCS365 *ΔspuC* and *ΔmorA* mutants do not have motility defects and form enhanced biofilms. Swimming motility assays (A) and crystal violet biofilm assays (B and C) were performed with WCS365 *ΔmorA* and *ΔspuC* mutants in (B) M63 media or (C) M63 media supplemented with 0.4% l-arginine. The *lapA*::Tn*5* mutant was used as a control for a biofilm-impaired mutant. Data represent averages of results from 4 to 5 biological replicates performed with 3 to 4 technical replicates per experiment. Letters designate levels of significance (*P* < 0.05) as determined by ANOVA and Tukey’s HSD.

Cyclic diguanylate (c-di-GMP) is a positive regulator of biofilm formation and negative regulator of motility in many *Proteobacteria* (23). As a result, we predicted that MorA, a predicted diguanylate cyclase/phosphodiesterase, promotes biofilm formation by synthesizing c-di-GMP and that the *ΔmorA* mutant would have increased motility and decreased biofilm formation. Unexpectedly, we found that neither the *ΔmorA* mutant nor the majority of other *Pseudomonas* sp. WCS365 deletion mutants had increased swimming motility (Fig. 4A; see also Fig. S6A). We found that several mutants had subtle decreases in surfing motility (24) (Fig. S6B). Deletion of the *gtsB* ABC transporter gene resulted in consistently impaired surfing and swimming motility (Fig. S6A and B). Collectively, these data indicate that increased bacterial motility does not underlie the rhizosphere fitness defect or induction of defenses in these mutants.

10.1128/mBio.00433-18.6 FIG S6 Biofilm and motility phenotypes of *Pseudomonas* sp. WCS365 mutants. (A) Swimming and (B) surfing motility assays with WCS365 mutants. All data were normalized for the wild-type control of a given experiment. Representative images of the plates that were used for quantification are shown below each bar graph. (C and D) Crystal violet assays were performed in (C) minimal M63 media or (D) M63 salts supplemented with 0.4% l-arginine. (A and B) Data represent averages of results from 3 biological replicates performed with 3 technical replicates per experiment. (C and D) Data shown represent averages of results from 3 biological replicates performed with 8 technical replicates per experiment. In panels A to D), asterisks (*) or letters designate significant differences (*P < *0.05 by ANOVA and Tukey’s HSD). Download FIG S6, PDF file, 0.2 MB. Copyright © 2018 Liu et al. 2018 Liu et al. This content is distributed under the terms of the Creative Commons Attribution 4.0 International license.

MorA has both predicted diguanylate cyclase (DGC) and phosphodiesterase (PDE) domains. DGC domains can positively regulate biofilm formation by promoting c-di-GMP accumulation, while PDEs can decrease biofilm formation and promote dispersal by lowering c-di-GMP levels (25). As a result, we tested whether the *ΔmorA* mutant and the remaining WCS365 mutants had alterations in biofilm formation in a standard *in vitro* crystal violet assay in M63 minimal media or M63 salts supplemented with arginine, which has previously been shown to enhance biofilm formation in *Pseudomonas* spp. (26, 27). We found that both the *ΔmorA* and *ΔspuC* mutants formed strongly enhanced biofilms in M63 media and that *ΔuvrA* and *ΔwapA* mutants formed weakly enhanced biofilms (Fig. 4B; see also Fig. S6C). Additionally, we found that only the Δ*spuC* mutant formed enhanced biofilms in M63 supplemented with arginine (Fig. 4C; see also Fig. S6D). SAD-51, a known surface attachment deficiency mutant of *Pseudomonas* sp. WCS365 with a transposon insertion in *lapA* (10), was used as a control. Enhanced biofilm formation by *Pseudomonas* sp. WCS365 *ΔmorA* and *ΔspuC* mutants *in vitro* suggests that hyperbiofilm formation or an inability to disperse may underlie their fitness defects in the rhizosphere.

### The phosphodiesterase activity of *Pseudomonas* sp. WCS365 MorA inhibits biofilm formation and is required for rhizosphere fitness.

**Fig 5:**
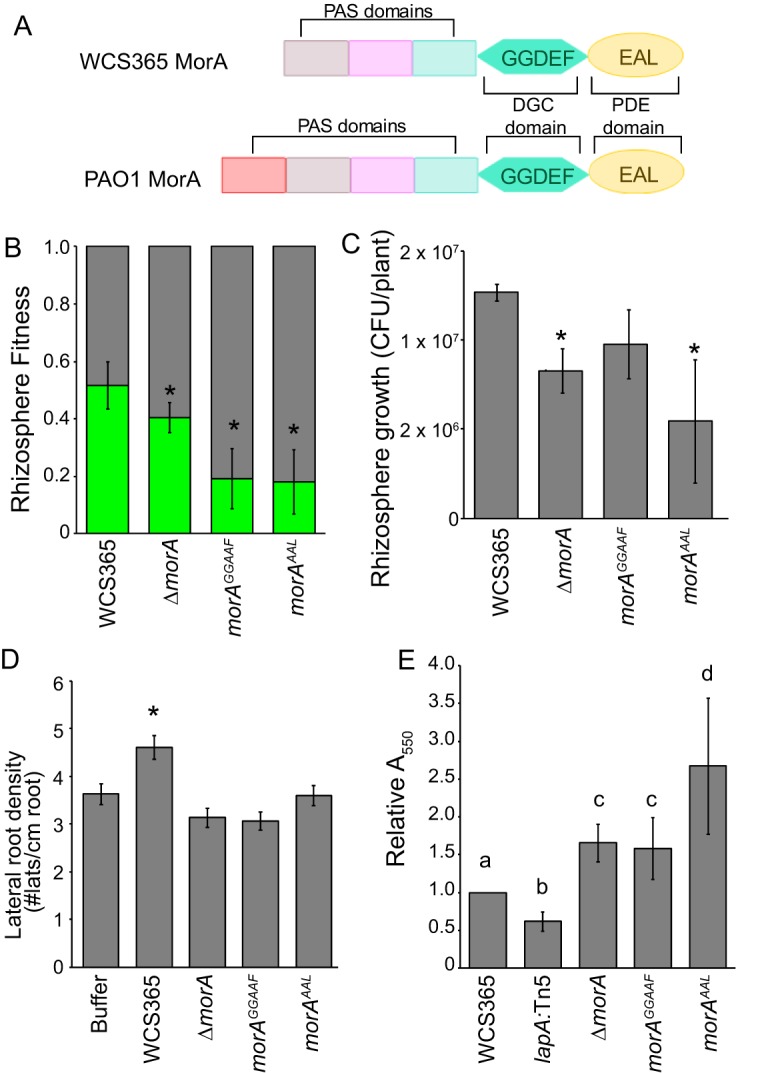
*Pseudomonas* sp. WCS365 *morA* acts as a phosphodiesterase to increase rhizosphere fitness and decrease biofilm formation. (A) Illustration of the functional domains of the MorA homolog in *Pseudomonas* sp. WCS365 and P. aeruginosa PAO1. Strains containing point mutations in the GGDEF or EAL motifs that inactivate the diguanylate cyclase domain (*morA^GGAAF^*) and the phosphodiesterase domain (*morA^AAL^*) were tested for (B) rhizosphere fitness, (C) rhizosphere growth, (D) growth promotion, and (E) biofilm formation. (B to D) *, *P < *0.01 (by Student’s *t* test); data represent averages of results from 3 biological replicates performed with at least 8 plants per replicate. (E) Letters designate levels of significance (*P <* 0.05 [by ANOVA and Tukey’s HSD]); data represent averages of results from 4 biological replicates performed with 8 technical replicates per experiment.

To test if the *morA* diguanylate cyclase and phosphodiesterase activities are necessary for rhizosphere fitness, we generated point mutations in the conserved GGDEF and EAL motifs to inactivate the diguanylate cyclase (*morA^GGAAF^*) and phosphodiesterase (*morA^AAL^*) domains (Fig. 5A). Surprisingly, we found that both the *morA^GGAAF^* and *morA^AAL^* mutants retained defects in rhizosphere growth and fitness as well as plant growth promotion (PGP) (Fig. 5B to D). We found that the *morA^AAL^* mutant showed even greater biofilm formation ability in a crystal violet assay and that *morA^GGAAF^* retained the enhanced biofilm formation ability of the *ΔmorA* mutant (Fig. 5E). That the *morA^GGAAF^* mutant retained the *ΔmorA* phenotype suggests that the conserved GGDEF sequence in fact contributes to phosphodiesterase activity, possibly by allosterically regulating the EAL domain activity (28). Collectively, these data suggest that *Pseudomonas* sp. WCS365 MorA acts as a phosphodiesterase to temper biofilm formation or promote dispersal in the rhizosphere.

### Putrescine acts as a signaling molecule to promote *Pseudomonas* sp. WCS365 biofilm formation.

**Fig 6:**
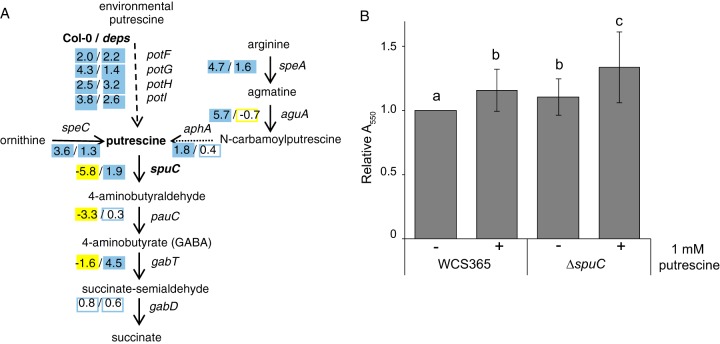
Putrescine promotes biofilm formation in *Pseudomonas* sp. WCS365. (A) Putrescine uptake, synthesis, and metabolism pathway in *Pseudomonas* sp. WCS365, with log_2_ fold change (log_2_FC) fitness data from the Tn-Seq experiment shown. (B) Crystal violet assays were performed in M63 media or M63 media with 1 mM putrescine. Data shown represent averages of results from 6 biological replicates performed with 8 technical replicates per experiment. Letters designate *P < *0.05 (by ANOVA and *t* tests).

Putrescine is present in the tomato rhizosphere (29), and so we wondered if putrescine could serve as a signaling molecule to promote bacterial biofilm formation. The *ΔspuC* mutant formed enhanced biofilms in the presence of arginine (Fig. 4C). Arginine can be converted to putrescine, and SpuC catalyzes the first step of putrescine catabolism (30); in the presence of arginine, an *spuC* mutant should overaccumulate putrescine (Fig. 6A). We confirmed that the *Pseudomonas* sp. WCS365 *ΔspuC* was unable to grow on putrescine as a sole carbon source (Fig. S7), indicating that it is unable to catabolize putrescine. We tested whether putrescine could directly promote biofilm formation in wild-type *Pseudomonas* sp. WCS365 and the *ΔspuC* mutant and found that putrescine is sufficient to promote biofilm formation in wild-type bacteria (Fig. 6B). Furthermore, the putrescine-mediated biofilm enhancement is exacerbated in the *ΔspuC* mutant (Fig. 6B), indicating that putrescine is a positive regulator of biofilm formation.

10.1128/mBio.00433-18.7 FIG S7 The *Pseudomonas* sp. WCS365 *ΔspuC* mutant cannot use putrescine as a sole carbon source. (A and B) Growth of the wild-type strain and the *ΔspuC* mutant in M9 plus 25 mM succinate (A) or M9 plus 25 mM putrescine (B). Download FIG S7, PDF file, 0.7 MB. Copyright © 2018 Liu et al. 2018 Liu et al. This content is distributed under the terms of the Creative Commons Attribution 4.0 International license.

If putrescine serves as a signaling molecule in the rhizosphere, other genes involved in putrescine synthesis or metabolism should also have had fitness defects in our experiment. We reconstructed the putrescine uptake, synthesis, and utilization pathways based on what is known in WCS365 and other organisms (30–32) (Fig. 6A). We overlaid our Tn-Seq fitness data onto the putrescine uptake, synthesis, and utilization pathway and found that insertions in *spuC*, *pauC*, and *gabT*, which are involved in the conversion of putrescine to succinate, all increase fitness in the rhizosphere of wild-type but not immunocompromised plants (Fig. 6A). Interestingly, we found that all genes potentially involved in putrescine uptake or synthesis had increased fitness scores in our Tn-Seq experiment, indicating that they are negative regulators of fitness (Fig. 6A). These data are inconsistent with putrescine being a significant carbon source in the rhizosphere; if it were, we would predict that a loss of uptake or synthesis would impair fitness in the rhizosphere. These data collectively support the hypothesis that putrescine may serve as a signaling molecule to trigger a *Pseudomonas* sp. WCS365 lifestyle change in the rhizosphere.

## DISCUSSION

Here we report a screen that identified a Pseudomonas fluorescens WCS365 putrescine aminotransferase (SpuC) and a phosphodiesterase (MorA) that are required to evade plant defenses. Deletion of either gene results in induction of pattern-triggered immunity (PTI) in *Arabidopsis* as measured by *FLS2*/*BAK1*-dependent inhibition of plant growth and in increased induction of the MAMP-inducible *MYB51* gene (Fig. 3). Previous studies have found that *Pseudomonas* spp. induce a subset of plant PTI responses while suppressing others (2, 3). Collectively, these data reveal novel mechanisms used by *Pseudomonas* sp. to avoid detection by a plant host.

A previous study found that an insertion between *spuC* and the *potFGHI* uptake system in WCS365 increased putrescine uptake and decreased rhizosphere fitness (29). In Pseudomonas aeruginosa, SpuC is involved in the catabolism of putrescine into 4-aminobutyraldehyde (30); because the *Pseudomonas* sp. WCS365 *ΔspuC* mutant cannot utilize putrescine (see Fig. S7 in the supplemental material), it may accumulate putrescine or related compounds. We found that arginine and putrescine promote biofilm formation in wild-type *Pseudomonas* sp. WCS365 and that loss of the *spuC* gene further enhances the biofilm-promoting effects of arginine and putrescine (Fig. 4C and 6). Putrescine is a positive regulator of biofilm formation in Yersinia pestis (33); while it is possible that the fitness defect in the *ΔspuC* mutant is due to an inability to metabolize putrescine as a sole carbon source, we propose instead that putrescine serves as a signal that informs *Pseudomonas* sp. of the presence of a eukaryotic host such as a plant. This in turn triggers a bacterial lifestyle switch to promote attachment and biofilm formation. Loss of the *spuC* gene results in hypersensitivity to exogenous putrescine and in changes in bacterial physiology that ultimately trigger plant defenses. Collectively, these data suggest that either arginine or putrescine in the plant rhizosphere might act as a signaling molecule to trigger a lifestyle change and evade plant defenses.

By performing an in-depth characterization of the role of a *Pseudomonas* sp. WCS365 diguanylate cyclase/phosphodiesterase A (DGC/PDEA) gene, *morA*, in rhizosphere competence and biofilm formation, we determined that MorA primarily acts as a phosphodiesterase to temper biofilm formation in the rhizosphere (Fig. 5). Interestingly, the *ΔmorA* mutant triggered *FLS2*-dependent defenses without changes in motility. This suggests that increased contact with the plant due to hyperbiofilm formation coupled with normal flagellin production may trigger host immunity. A similar phenomenon was observed in P. aeruginosa; increased exopolysaccharide production facilitates a flagellin-induced, TLR5-mediated inflammatory response in human epithelial cells due to increased adherence and contact between the bacteria and cells (34). Bacterial DGC/PDEA activities regulate the intracellular levels of the bacterial second messenger cyclic diguanylate (c-di-GMP). In P. aeruginosa, DGCs promote c-di-GMP synthesis and positively regulate biofilm formation whereas subsequent lowering of c-di-GMP synthesis by PDEAs downregulates biofilm production and promotes dispersal (25). Because *morA* acts as a PDEA, this suggests that its role may be to temper biofilm formation and/or promote dispersal in the rhizosphere. These data suggest that the ability to disperse or downregulate biofilm production may be required to evade induction of host defenses.

In mixed inoculations, we observed that plant defenses were targeted only toward specific mutant bacteria and not toward the community as a whole. We used two mixed-inoculation strategies: a Tn-Seq community and 1:1 inoculations of wild-type and mutant bacteria. With the Tn-Seq library, we observed similar levels of colonization by the library and wild-type bacteria, which indicates that any defense response that was effective at killing bacteria was highly localized. The second mixed-inoculation strategy was the use of a 1:1 ratio of wild-type or mutant bacteria. Under these conditions, we did observe a slight but not statistically significant decrease in the total number of bacteria. This again indicates that any defense responses must be highly localized at the level of one cell or a group of cells rather than the entire plant. Collectively, these data suggest a model where a highly localized defense response may help target a pathogen invader without disrupting the complex and diverse rhizosphere microbial community. As a result, we hypothesize that Δ*spuC* and Δ*morA* mutants are poor rhizosphere colonizers due to an inability to disperse from the initial site of colonization after triggering plant immune responses.

In summary, evasion or suppression of the plant immune system is essential for the ability of pathogens to successfully infect their plant hosts. Our results support a growing body of evidence indicating that avoiding plant defenses is also critical for survival of commensals in association with a host (35). Many studies have pointed to attachment being critical for virulence of bacterial pathogens (36) and colonization of commensals (37). However, our work shows a positive correlation between hyperformation of biofilm and induction of plant defenses. This work indicates that changes in bacterial physiology may be necessary for evasion of plant defenses and survival in association with a eukaryotic host.

## MATERIALS AND METHODS

### Plant growth.

For the Tn-Seq screen, seeds were sterilized using chlorine gas, which successfully eliminates endophytes detectible by 16S rRNA sequencing (9). For growth promotion and root colonization experiments, seeds were sterilized using either chlorine gas (exposure to 100 ml bleach plus 3 ml concentrated hydrochloric acid in an air-tight container for 4 h) of bleach sterilization (70% ethanol for 2 min followed by 10% bleach for 5 min followed by three washes in sterile distilled water). Plants were grown under day/night conditions (16 h/8 h) at 22°C under 100 µE light.

*Arabidopsis* genotypes were in the Col-0 background and included the following: *dde2-1*, *ein2-1*, *pad4-1*, and *sid2-2*; *“deps”* mutant (Tsuda et al. [15]); *bak1-4* (38); *fls2* (22); and *MYB51pro-GUS* (2).

### Strains, media, and culture conditions.

*Pseudomonas* sp. WCS365 was grown on Luria-Bertani (LB) agar or in LB medium at 28°C; Escherichia coli strains were grown on LB agar or in LB medium at 37°C. When appropriate, the following antibiotics were used at the indicated concentrations: 5 µg/ml gentamicin (E. coli) or 10 µg/ml (*Pseudomonas*), 50 µg/ml kanamycin (Kan), or 15 µg/ml nalidixic acid.

### Genome sequencing of *Pseudomonas* sp. WCS365 and phylogenomic analysis.

Bacterial DNA was isolated using Qiagen Puregene kit A and sonicated into ∼500-bp fragments. Library construction and genome assembly were performed as described previously (39, 40). The draft genome of the *Pseudomonas* sp. was assembled into 60 contigs containing 6.56 Mb and a predicted 5,864 coding sequences. The WCS365 Whole-Genome Shotgun project data have been deposited at DDBJ/ENA/GenBank under accession no. PHHS00000000. The version described in this paper is version PHHS01000000.

### Tn-Seq library preparation.

A mariner transposon (pSAM_DGm [41]) was introduced in *Pseudomonas* sp. WCS365 via conjugation with SM10λpir E. coli. We found that the transposon integrated into the E. coli genome with detectible frequency, and so we minimized the culture growth time to ∼6 h. The conjugation was left at 28°C for 2 h to minimize replication and the occurrence of sibling mutants. The conjugation was then scraped off plates and frozen. The mating mix was plated on LB in 100 mM petri dishes with gentamicin (10 μg/ml) and nalidixic acid (15 μg/ml) at a density of 1,500 to 3,000 colonies per plate. After we approximated the number of colonies, they were scraped off the plates and pooled to ∼10,000 colonies (4 to 6 plates) per mix. This was repeated 10 independent times from 10 mating mixes for a total of an estimated 100,000 colonies. The optical density at 600 nm (OD_600_) of each independent pool was measured, and, to make the final pool, the density was normalized such that approximately the same number of cells was added from each colony. The library was diluted to an OD_600_ of 0.2 and allowed to recover for 1 h in LB prior to aliquoting and freezing. An aliquot of the library was plated, and CFU were counted before the final plant inoculation step.

We sequenced the region flanking the transposon insertions in our library (Materials and Methods; see also Fig. S3 in the supplemental material). We mapped the insertion sites to the 6.56-Mb draft *Pseudomonas* sp. WCS365 genome and found that the library contained insertions in 66,894 TA dinucleotide sites distributed across the genome, with approximately 9.8 insertions per 1,000 bp. Among the 5,864 annotated genes in WCS365, we identified insertions in 5,045 (86%). The distribution of insertions by gene is shown in Fig. S3B, and the gene list is presented in Data Set S1 in the supplemental material; we found a mean of 10.3 insertions per gene and a median of 8 insertions.

### Tn-Seq experimental setup and sequencing methods.

Sterile plant growth substrate was prepared by mixing 2 parts Turface Prochoice calcine clay, 2 parts Turface QuickDry, and 1 part perlite. The mixture was washed 10 times with distilled water to remove soluble nutrients. Plant tissue culture vessels (C1775; Phytotechnology Laboratories) (12 cm in diameter) were filled to a depth of 3 cm with the mixture, ensuring that the mixture was saturated with water but that water did not pool in the box. Hoagland’s solution (10 ml) was added to each box (Fig. S1). The result was a porous growth substrate. Boxes were capped and autoclaved.

To facilitate *Arabidopsis* germination, twenty plugs of mannitol salt (MS) agar (1× with 2% sucrose) (3 mm^3^) were evenly distributed on the growth medium surface and seeds were sowed directly on the agar plugs (Fig. S1). A total of 10 boxes per treatment were used with 20 plants per box (*n* = 200 plants), and three biological replicates were used per treatment. Treatments included Col-0, the immunocompromised *deps* mutant, and a no-plant control (20 mM succinate was added on top of the plugs in lieu of plants as a bacterial nutrient source). Plants were grown for 2 weeks prior to inoculation. The library was diluted to 5 × 10^4^ CFU/ml based on plate counts, and 200 μl was added to each plant or each control agar plug for a total of 10^4^ bacteria per plant. A total of 20 plants or no-plant equivalents were inoculated per box for a total of 2 × 10^5^ bacteria per box, and 2 × 10^6^ bacteria were sampled for the entire experiment; each insertion was represented about 20 times the original inoculum. We found that each plant could support a total of 5 × 10^7^ CFU/gram (Col-0) and that each plant weighed about 50 mg at the end of the experiment, meaning that our pool increased 250-fold (∼8 doublings) over the course of the experiment. Bacteria were allowed to grow for 1 week before harvesting was performed.

To harvest bacteria, plants were removed from the growth substrate and loose soil was removed (Fig. S1). DNA was isolated using a MoBio Power Soil DNA isolation kit (columns for up to 10 g of material); all materials from a single treatment and replicate were processed together. Yields were on the order of 3 to 12 µg of DNA from the plant and clay samples, and 3 µg of DNA was used as an input for library construction.

Sequencing libraries were prepared as described previously (42) using cleavage with the MmeI enzyme with modifications. Adapter and primer sequences, along with a schematic of library construction, can be found in Fig. S3. Sequencing libraries include 3 replicates of (i) the input library, (ii) the Col-0 rhizosphere, (iii) the hormone mutant (*dde2-2*, *ein2-1*, *pad4-1*, *sid2-2*), and (iv) no plant treatments. Each replicate (12 samples total) was indexed separately. Sequencing libraries were prepared by digesting input DNA with the MmeI enzyme, end repairing, and ligating a double-stranded blunt-ended adapter molecule. Transposon- and adapter-specific primers were used to amplify the region flanking the transposon (the transposon is palindromic, and so the two directions should amplify with similar frequencies). The presence of a predicted 169-bp product was confirmed with an Agilent Bioanalyzer. All 12 samples were pooled and run in the same Illumina HiSeq lane using single-end 50-bp reads.

### Tn-Seq data analysis.

Data analysis was performed using Galaxy and a modified version of the MaGenTA pipeline described previously (18). Our custom adapters and barcodes are shown in Data Set S1, and a schematic of library construction is shown in Fig. S3. The adapter was trimmed using the custom sequence 5′-ACAGGTTGGATGATAAGTCCCCGGTCT-3′. Sequencing reads were trimmed to remove the transposon sequencing, and 21 to 22 bp that represented the flanking region post-MmeI cleavage remained. After barcode splitting and trimming were performed, between 458,679 and 1,298,597 reads were assigned to each individual treatment. Sequences were mapped back to the *Pseudomonas* sp. WCS365 draft genome using Map_with_Bowtie_for_Illumina and the following custom settings: –*n* = 1 (one mismatch allowed), −1 = 15 (15 bp seed), −y = try hard, and –m = 1.

We detected 66,893 unique TA insertion sites in our input library. We observed a significant bottleneck in all plant and clay treatments corresponding to average losses of 38%, 35%, and 33% of the insertions in the Col-0, immunocompromised *deps*, and clay samples, respectively. The MaGenTA fitness calculations pool all insertions per gene before calculating fitness. Using this approach, we found that all but 192 (3%) of the genes with insertions in the in the input retained insertions in the Col-0, *deps*, and clay samples.

Genes were considered to significantly affect fitness if insertions in them resulted in an average log_2_(5)-fold increase or decrease in fitness and they had a *P* value of <0.05 (Data Set S1). To further study genes with large differences in the levels of fitness between Col-0 and the *deps* rhizospheres, we looked at the gene set that corresponded to a greater than −log_2_(10)-fold difference between the Col-0 and *deps* fitness scores once each was normalized to the clay-only control. We considered only genes with a normalized (rhizosphere/clay) fitness score of less than −log_2_(3) for Col-0 and of greater than log_2_(3) for the immunocompromised *deps* mutant.

### Strain construction.

Primers used for site-directed mutagenesis are listed in Data Set S1.

Deletion strains *Pseudomonas* sp. WCS365 *ΔmorA*, *ΔkatB*, *ΔcolR*, *ΔwapA*, *ΔgtsB*, and *ΔuvrA* were constructed by amplifying 500 to 700 bp of the upstream and downstream regions flanking the open reading frame and using overlap extension PCR (43) to join the two pieces prior to ligation into the pEXG2 vector (44). The pEXG2 vector confers gentamicin resistance and contains the *sacB* gene for counterselection on sucrose. After the correct insertion was confirmed by sequencing, the plasmid was transformed into SM10λpir. Conjugations were performed with *Pseudomonas* sp. WCS365 by mixing a 2:1 ratio of washed overnight cultures of WCS365 with SM10λpir and the desired plasmid, spotting onto King’s B plates, allowing the mating spots to dry, and incubating for 4 h at 28°C. Mating mixes were then scraped off the plates and plated on selective media with 10 µg/ml gentamicin and 15 µg/ml. Successful integration of the plasmid into the genome confirmed by patching candidate colonies on sucrose or gentamicin. A second crossover event was selected by growing colonies overnight in media without selection and then plating sucrose without antibiotics. Candidate colonies were screened using primers outside the initial construct, and the final construct was confirmed by sequencing.

Deletion strains *Pseudomonas* sp. WCS365 Δ*spuC* and *ΔcioA* were constructed using a three-way cloning strategy. First, flanking regions of each gene were amplified using primers with added terminal restriction sites. The exterior ends of the regions were each modified with a unique restriction site, whereas a third restriction site was used for the interior ends of both regions. The suicide vector (pNPTS138) was then digested using the enzymes for the exterior ends, and each region was digested using the two enzymes appropriate for its own modified ends. The digested vector and the two flanking regions were then ligated and transformed into E. coli, followed by plasmid isolation and sequencing to ensure the integrity of the inserted deletion allele. pNPTS138 is a suicide vector developed for use in the *Alphaproteobacteria* Caulobacter crescentus (M. R. K. Alley, unpublished data). Because it has a ColE1 origin which is specific to the Enterobacteriaceae, it should function as a suicide vector in *Pseudomonas*, which we confirmed by performing conjugations with an empty vector. Conjugations were carried out by mixing 1 ml of wild-type WCS365 with 1 ml of the WM3064 E. coli DAP (diaminopimelic acid) auxotroph strain carrying a suicide vector and plating 10 µl of the washed and concentrated cell mixture on LB supplemented with 0.3 mM DAP. After 4 to 6 h, the “mating spot” was resuspended in 1 ml of supplement-free LB and dilutions were plated on LB with 50 mg/liter kanamycin (LB-Kan). Kanamycin-resistant WCS365 clones were restreaked on LB-Kan to purify and then patched densely onto no-salt LB with 10% sucrose to grow overnight as a lawn, which we then restreaked for single colonies. This was necessary because the *sacB* locus present on pNPTS138 did not confer strong sucrose sensitivity. This may have been due to low expression of *sacB* in *Pseudomonas*, as Rietsch et al. developed the suicide vector pEXG2 for P. aeruginosa by adding a strong promoter to drive *sacB* expression (44). Nevertheless, 5% to 10% of the single colonies grown on sucrose media were kanamycin sensitive, indicating that there may have been weak sucrose counterselection. These Kan sensitive (Kan^S^) colonies were screened using PCR with the exterior primers for the flanking regions to distinguish strains with the deleted allele from wild-type revertants.

Site-directed mutagenesis of *Pseudomonas* sp. WCS365 *morA* GGDEF domain (Fig. 5A) was performed by amplifying *Pseudomonas* sp. WCS365 *morA* with FL05 and FL06 or with FL07 and FL08 and joining the product by overlap extension (43). Similarly, the EAL domain was mutagenized by joining the product amplified by FL01 and FL02 and by FL03 and FL04. The joined PCR product was digested and ligated to pEXG2 vector for integration of WCS365 (44). Genomic mutations were confirmed by Sanger sequencing. D928AE929A mutations were introduced to the GGDEF domain (*morA^GGAAF^*), and an E1059A mutation was introduced into the EAL domain (*morA^AAL^*). These mutations were designed to abolish the catalytic activities of diguanylate cyclase and phosphodiesterase, respectively (45–48). Screening of colonies to identify those with the correct mutations was performed using SNAP primers (49) designed to amplify the wild-type or mutant alleles (Data Set S1).pSMC21 (*Ptac-GFP*) and pCH216 (*Ptac-mCherry*) were transformed into wild-type or mutant *Pseudomonas* sp. WCS365 strains by pelleting an overnight culture, washing with 300 mM sucrose, and electroporating at 2.5 kV, 200 ohms, and 25 μF. Transformants were selected on LB with 50 µg/ml kanamycin. pCH216 was generated from pSMC21 (50) by excising GFP via a partial digestion with XbaI and PstI and replacing it with PCR-amplified mCherry ligated into the XbaI and PstI sites.

### Annotation of candidate genes.

WCS365_04639 was annotated as a catalase gene. PaperBLAST (http://papers.genomics.lbl.gov/cgi-bin/litSearch.cgi) results suggested that its product was highly similar to *Pseudomonas* sp. SWB25 protein KatB (90% identity, 100% coverage) and to P. aeruginosa PAO1 protein KatB (81% identity, 95% coverage).

The WCS365_05664 gene product is similar to the previously characterized P. aeruginosa PAO1 protein MorA, a known diguanylate cyclase/phosphodiesterase (68% identity, 99% coverage) (51, 52).

WCS365_00305 was originally annotated as a putative aminotransferase gene. BLAST results suggested that WCS365_00305 encoded an aspartate aminotransferase; however, the most similar gene product (as determined using PaperBLAST) was SpuC (encoded by PA0299), a putrescine aminotransferase in P. aeruginosa PAO1.

Based on annotation, WCS365_05132 encodes a UvrABC system protein A, consistent with protein BLAST and PaperBLAST results. Its homolog *uvrA* in Escherichia coli encodes a subunit of the ABC excinuclease responsible for the repair of UV-induced thymine dimer and other DNA damage (53, 54).

WCS365_04646 was annotated as a cytochrome *bd*-I ubiquinol oxidase subunit 1 gene, consistent with PaperBLAST results. This gene is highly similar to the previously characterized P. aeruginosa PAO1 gene *cioA* (79% identity, 98% coverage), which, together with the gene product of *cioB*, forms CIO, a cytochrome with low affinity to oxygen (55).

The sequence of the WCS365_04136 gene product is highly similar (97% identity and 100% coverage) to the predicted amino acid sequence of *Pseudomonas* sp. SBW25 *gtsB* (PFLU4845), which has been shown to function as a glucose permease subunit of an ATP-binding cassette transporter (56).

Nucleotide BLAST result suggested that WCS365_05599 encodes a type 6 secretion system (T6SS)-dependent secreted Rhs protein. Rhs was known to be a contact-dependent toxin delivered to neighboring bacterial cells, causing growth inhibition (57). However, no T6SS-related genes, such as *vgrG* or *hcp* genes, were found in the same contig as WCS365_05599 (58). Hence, we surmise that WCS365_05599 encodes a distantly related, T6SS-independent, contact-dependent toxin, WapA (57).

We reconstructed the putrescine uptake, synthesis, and utilization pathways based on what is known in WCS365 and other organisms (30–32) (Fig. 6A). We identified putrescine uptake system operon *potFGHI* (WCS_00300-WCS_00304), a gene involved in conversion of arginine to putrescine (*speA* WCS365_02314; *aguA* WCS365_01963; *aphA* WCS365_00490). Notably, we were unable to identify a homologue of P. aeruginosa *aguB* (acts with *aphA* to catalyze the conversion of N-carbamoylputrescine to putrescine [59]) in the WCS365 genome or in the genome of its close relative *Pseudomonas* sp. NFM421. This indicates that *Pseudomonas* sp. WCS365 cannot convert arginine to putrescine or that *aphA* alone catalyzes this reaction or that a different enzyme substitutes for *aguB*. For genes involved in conversion of putrescine to succinate, we identified *pauC*, *gabT*, and *gabT* homologues in WCS365 (WCS365_03989, WCS365_05732, and WCS365_05733).

### Rhizosphere and *in vitro* bacterial growth and fitness assays.

Bacterial growth in the rhizosphere was quantified by growing *Arabidopsis* in 48-well clear-bottom plates with the roots submerged in hydroponic media and the leaves separated by a Teflon mesh disk (9). Plants were inoculated with wild-type and/or mutant *Pseudomonas* sp. WCS365 strains containing plasmid pSMC21 (*pTac-GFP*) or plasmid pCH216 (*pTac-mCherry*), and bacterial fluorescence was read with a SpectraMax i3x fluorescence plate reader (Molecular Devices) (481/515 GFP; 577/608 mCherry) (9). Briefly, 9-mm-diameter sterile Teflon mesh disks (Macmaster Carr) were placed individually in 48-well tissue culture-treated plates (Falcon). Each well was filled with 300 µl 0.5× MS media plus 2% sucrose, and a single sterilized *Arabidopsis* seed was placed at the center of each disk. The medium was replaced with 270 µl 0.5× MS media with no sucrose on day 10, and plants were inoculated with 30 µl bacteria at an OD_600_ of 0.0002 (final OD_600_, 0.00002; ∼1,000 cells per well) on day 12. For fitness assays, 15-µl volumes each of the wild-type (mCherry) and mutant (GFP) strains were added to each well. To estimate the final relative proportion of each bacterial strain, standard curves relating fluorescence intensity to bacterial OD_600_ for each fluorophore in each mutant background were generated. The fluorescence signal for each plant was measured preinoculation, and this background value was subtracted from the final well readings. The standard curve was used to estimate CFU of each bacterial strain per well. The fraction that each strain contributed to the total bacterial population was determined. Data represent an average of at least 3 independent biological replicates per bacterial genotype, with a minimum of 6 wells used per bacterial strain per experiment.

Bacterial *in vitro* growth and fitness were measured with a SpectraMax i3x plate reader (Molecular Devices). Overnight cultures were diluted to an OD_600_ of 1 in 10 mM MgSO_4_. A 3-µl volume of diluted culture was added to 97 µl LB (rich media), M9 plus 30 mM succinate (minimal media), or root exudate (described below).

### *In vitro* bacterial growth and fitness.

Bacterial growth curves were performed by using bacterial cultures grown overnight in LB and then pelleted, washed in 10 mM MgSO_4_, and diluted to an OD_600_ of 1. A 3-µl volume of the culture was mixed with 97 µl of growth media for a starting OD_600_ of 0.03. Bacteria were grown in rich media (LB), minimal media (M9 salts plus 30 mM succinate), or root exudate (M9 salts plus 0.7× root exudate as the sole carbon source). Bacteria growth was quantified by measuring OD_600_ on a Versamax (Molecular Devices) plate reader. Doubling times were calculated using the exponential-growth stage for each experiment, and data reported represent averages of results from 3 biological replicates.

For bacterial growth in competition with the wild type, mutant strains expressing GFP were mixed in a 1:1 ratio with wild-type strains expressing mCherry. Red and green fluorescence as well as OD_600_ were measured for each well. Using a standard curve generated for each fluorophore for each mutant or wild-type strain, the approximate bacterial OD was calculated and plotted as mutant growth in competition with the wild type (9). For fitness measurements, the fraction of the well represented by each mutant relative to the total bacterial growth in the well was calculated. For each experiment, 3 to 4 technical replicates were performed and each growth curve was repeated at least 3 times. Doubling times were calculated using the exponential-growth stage for each experiment, and data reported represent averages of results from 3 biological replicates.

Root exudate was collected by growing plants in 48-well plates for 12 days in 0.5× MS media with 2% sucrose (9). The medium was replaced with 0.5× MS media with no sucrose and collected 1 week later. Exudate was pooled from multiple wells from 4 plates (∼200 plants). The final root exudate contained spent MS media as well as (potentially) trace amounts of sucrose left from the initial plant media.

### Plant growth promotion (PGP) assays.

The OD_600_ of *Pseudomonas* sp. WCS365 overnight cultures was measured before the cells were spun down at 10,000 × *g* for 3 min and washed with 10 mM MgSO_4_. After washing was performed, cells were resuspended and diluted to an OD_600_ of 0.01 in 10 mM MgSO_4._ Five-day-old *A. thaliana* seedlings on 0.5× MS plates were inoculated at the root tips with 1 µl of diluted cell suspension. Images of growth promotion plates were taken with an Epson V850 scanner. Root length was quantified using the “Measure” function in Image J, and lateral roots were counted manually using the scanned images.

### Histochemical GUS staining.

Seedlings were grown in 96-well plates in 100 µl 1× MS media with 2% sucrose as described previously (2). The medium was changed after 7 days, and bacteria were added to a final OD_600_ of 0.002 (20 µl OD_600_ = 0.01 per 80 µl media). β-Glucuronidase (GUS) staining solution was added 16 h later, and the reaction mixture was incubated at 2 h at 37°C. The GUS solution was removed, and seedlings were cleared with 95% ethanol overnight for imaging.

### Crystal violet biofilm assays.

Biofilm assays were performed as previously described (60, 61). Briefly, overnight cultures of *Pseudomonas* sp. WCS365 cells were spun down at 10,000 × *g* for 3 min and washed twice, resuspended, and diluted to an OD_600_ of 0.1 with M63 medium (1× M63 salt, 0.2% glucose, 0.5% Casamino Acids, 1 mM MgSO_4_), M63-putrescine medium (1× M63 salt, 0.2% glucose, 0.5% Casamino Acids, 1 mM putrescine, 1 mM MgSO_4_), or M63R (1× M63 salt, 0.4% l-arginine, 1 mM MgSO_4_) when appropriate. Diluted cultures (100 μl) were incubated at 27°C for 17 h in non-tissue culture-treated 96-well plates (Falcon; product no. 351177). After incubation, the plate was rinsed in distilled water twice before staining of the biofilm with 125 µl of 0.1% crystal violet for 10 min. Excess crystal violet was washed three times in distilled water, and the plate was dried overnight before solubilizing the crystal violet in 125 µl of 30% acetic acid for 10 min and then transferring to a new 96-well, flat-bottom plate for spectrophotometric reading. Absorbance was measured at 550 nm. Background signals were measured from wells containing 30% acetic acid and were subtracted from the absorbance reading. All average absorbance signals were normalized against the wild-type values.

### Motility assays.

Motility assays were performed as previously described (24, 62). Overnight cultures were spun down at 10,000 × *g* for 3 min, washed, resuspended, and diluted to an OD_600_ of 1.0 with M9S (1× M9 supplemented with 10 mM sodium succinate). For swimming motility, M9S 0.3% agar plates were inoculated by stabbing the plates with an inoculation needle dipped in the diluted culture without completely piercing the agar. Plates were incubated at 27°C for 65 h before imaging. For surfing motility, 0.3% agar M9S medium supplemented with 0.4% of citrus pectin (Alfa Aesar; catalogue no. J61021-22) was used. Plates were inoculated with 1 µl of diluted culture and incubated at 27°C for 24 h before imaging.

### Data availability.

The WCS365 Whole Genome Shotgun project data have been deposited at DDBJ/ENA/GenBank under accession PHHS00000000. The version described in this paper is version PHHS01000000.
