# Supplementary figures and images for: A Genome-Wide Screen Identifies Genes in Rhizosphere-Associated Pseudomonas Required to Evade Plant Defenses
Source: mBio. 2018 Nov 6;9(6):e00433-18. doi: 10.1128/mBio.00433-18 (PMC6222131; doi:10.1128/mBio.00433-18)

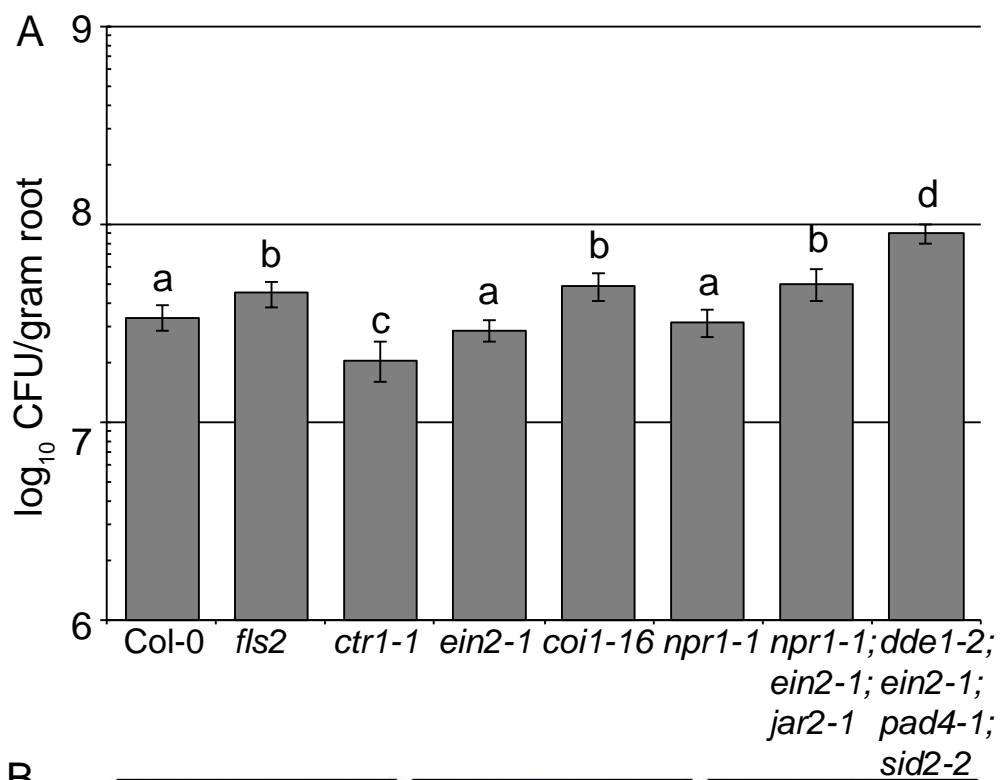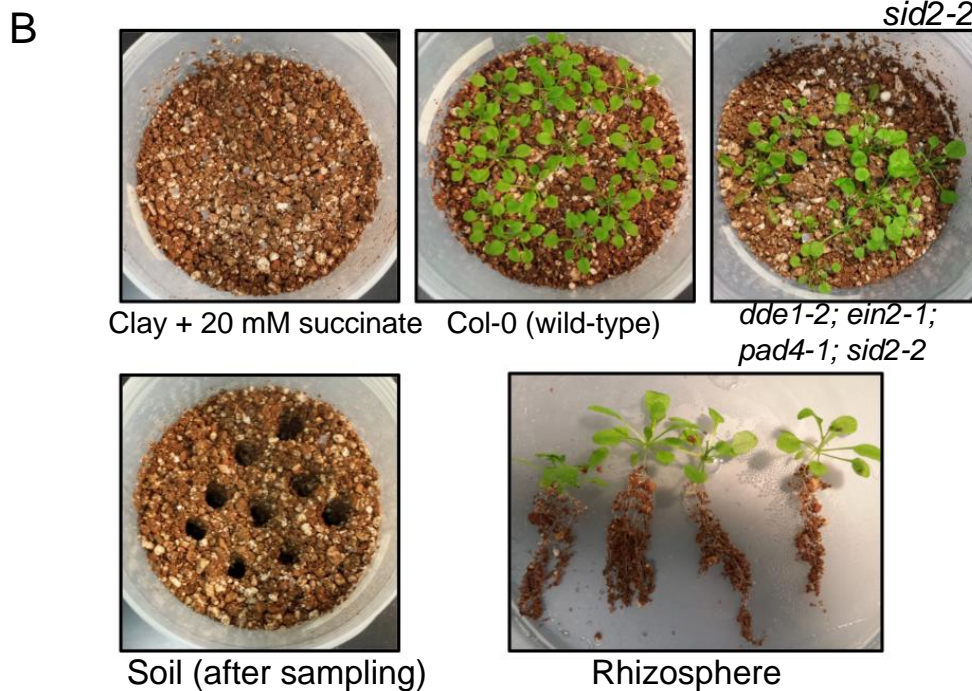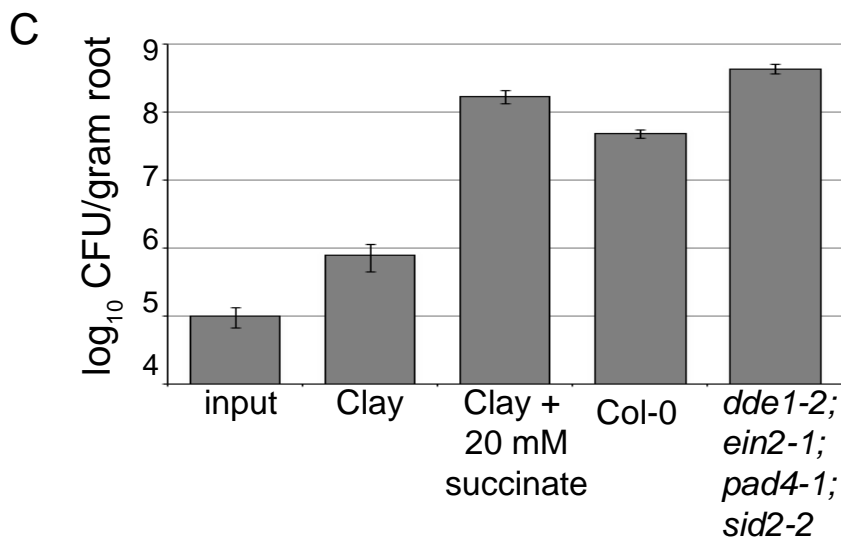

Supplement: FIG S1 [file mbo005184154sf1.pdf]

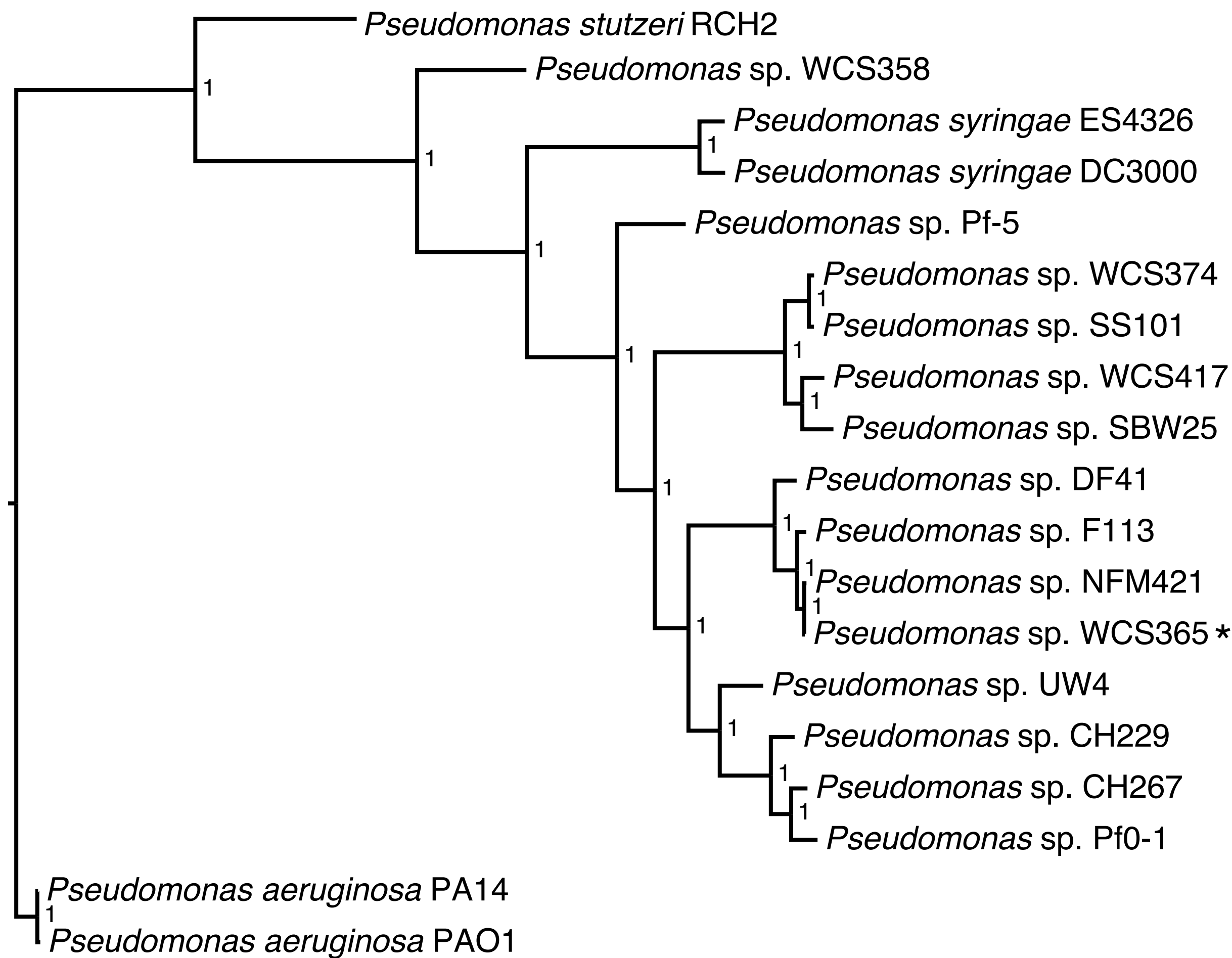

0.3

Supplement: FIG S2 [file mbo005184154sf2.pdf]

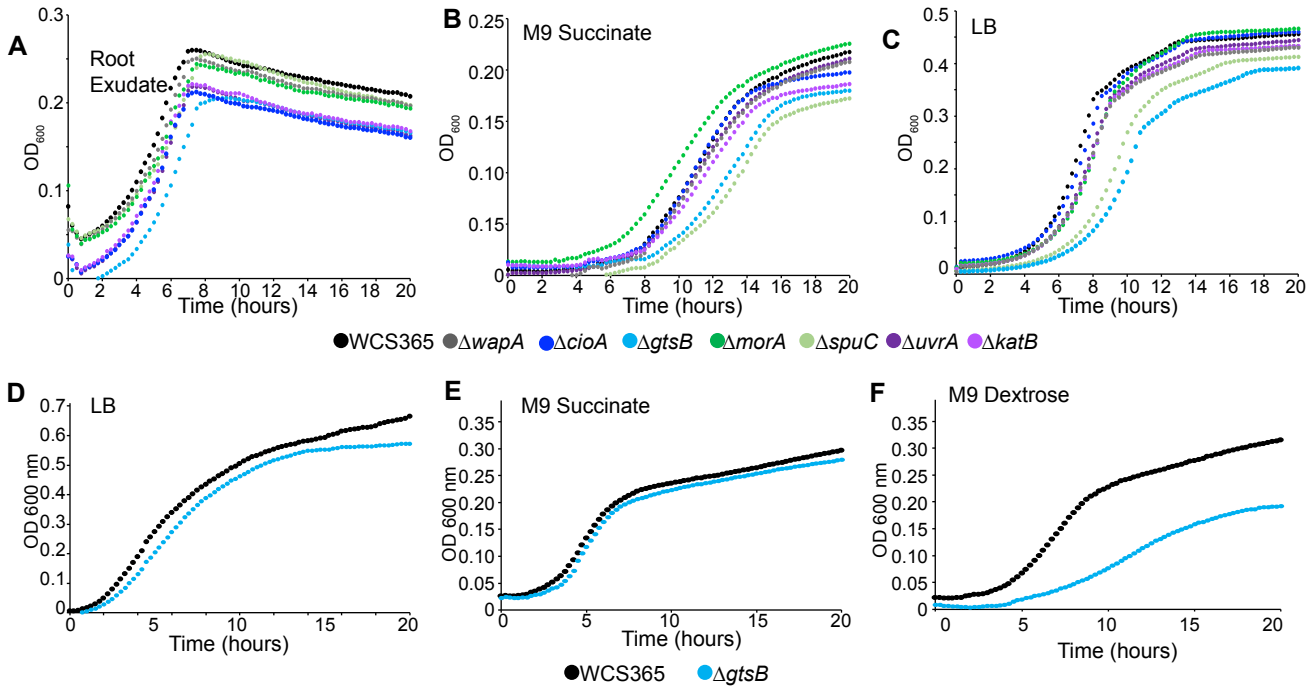

Supplement: FIG S4 [file mbo005184154sf4.pdf]

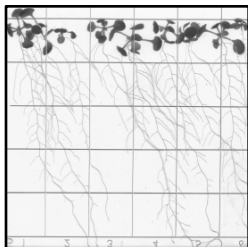

Buffer

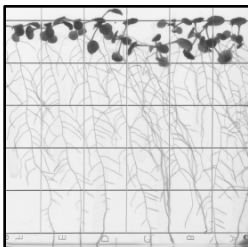

WCS365

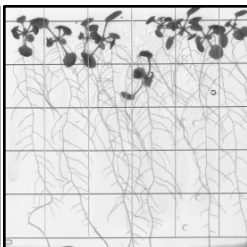

$\Delta wapA$

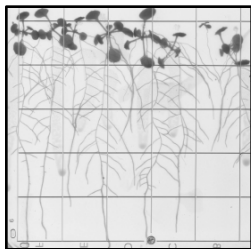

$\Delta cioA$

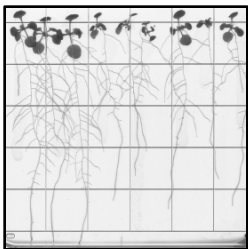

$\Delta gtsB$

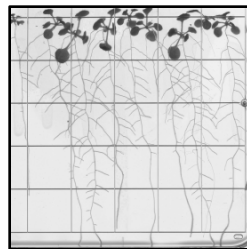

$\Delta morA^{**}$

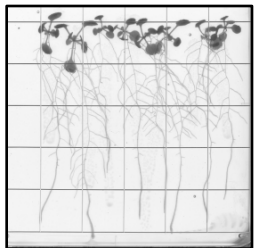

$\Delta spuC^{**}$

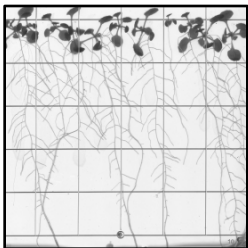

$\Delta uvrA$

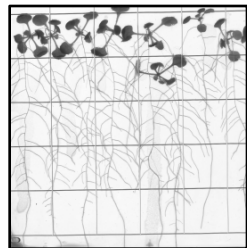

$\Delta katB$

Supplement: FIG S5 [file mbo005184154sf5.pdf]

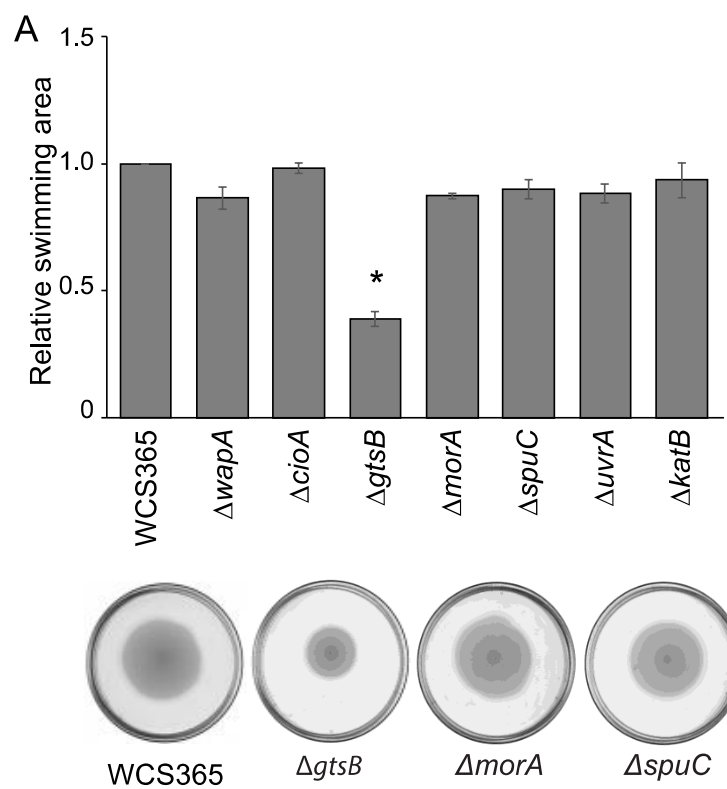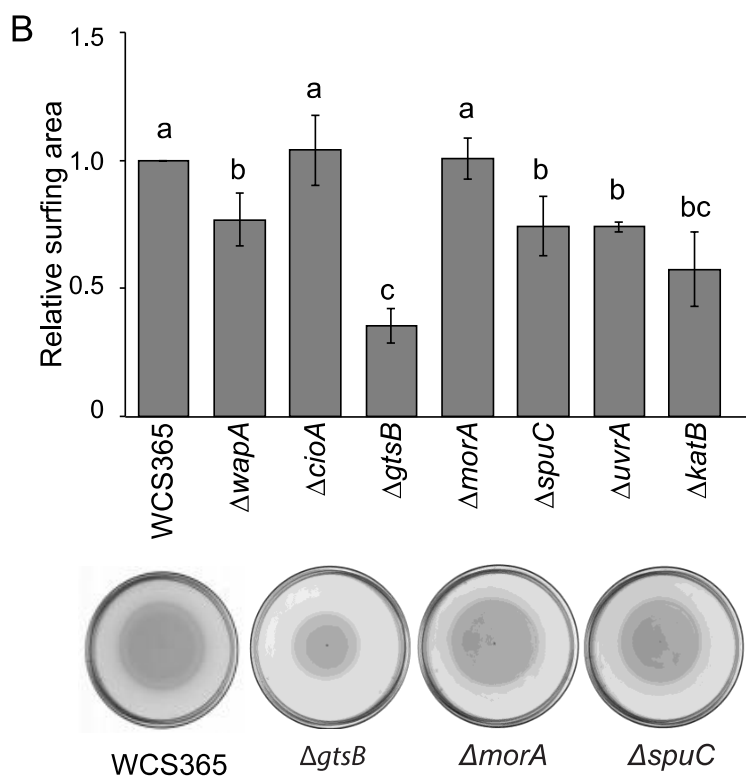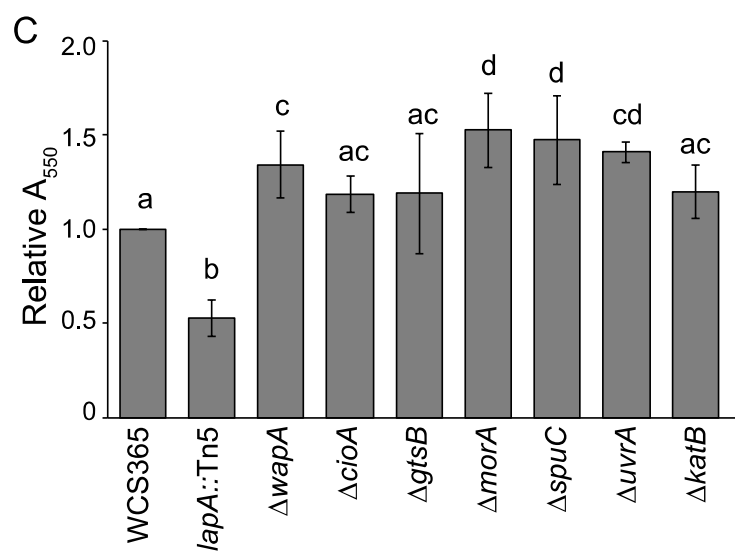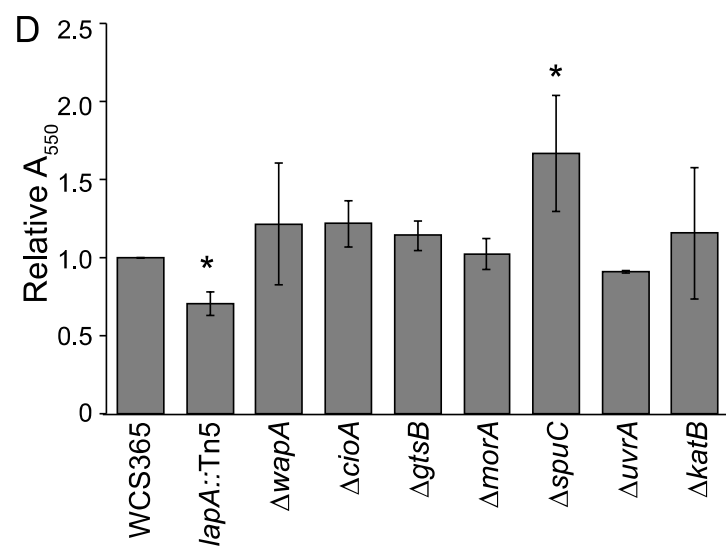

Supplement: FIG S6 [file mbo005184154sf6.pdf]

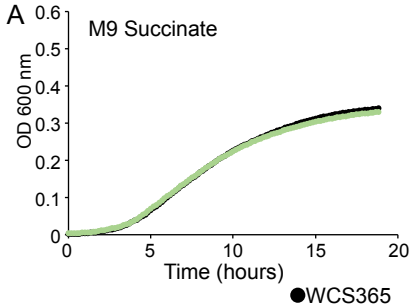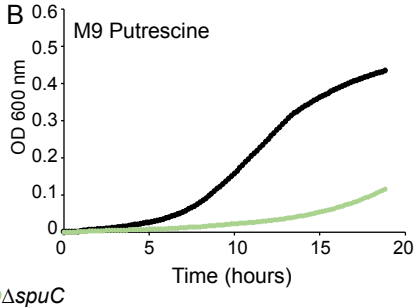

Supplement: FIG S7 [file mbo005184154sf7.pdf]
