# Supplementary material for: A Genome-Wide Screen Identifies Genes in Rhizosphere-Associated Pseudomonas Required to Evade Plant Defenses
Source: mBio. 2018 Nov 6;9(6):e00433-18. doi: 10.1128/mBio.00433-18 (PMC6222131; doi:10.1128/mBio.00433-18)
Supplement: TABLE S1 [file mbo005184154st1.docx]

|  | **Growth** | | | **In competition with WCS365** | | |
| --- | --- | --- | --- | --- | --- | --- |
|  | **LB** | **M9 succinate** | **Root Exudate** | **LB** | **M9 succinate** | **Root Exudate** |
| **WCS365** | 1.5 ± 0.1 | 2.0 ± 0.2 | 1.9 ± 0.3 | 1.4 ± 0.2 | 2.1 ± 0.3 | 1.9 ± 0.1 |
| ***∆colR*** | 1.6 ± 0.1 | 2.6 ± 0.6 | 1.8 ± 0.2 | 2.2 ± 0.8 | 2.4 ± 0.1 | 1.8 ± 0.3 |
| ***∆wapA*** | 1.5 ± 0.2 | 2.0 ± 0.3 | 2.2 ± 0.1 | 1.7 ± 0.1 | 2.1 ± 0.1 | 1.8 ± 0.2 |
| ***∆cioA*** | 1.3 ± 0.1 | 2.1 ± 0.1 | 1.8 ± 0.1 | 1.7 ± 0.2 | 2.3 ± 0.3 | 2.2 ± 0.3 |
| ***∆gtsB*** | 1.6 ± 0.1 | 2.2 ± 0.6 | 2.3 ± 0.8 | **3.2 ± 0.5**** | 2.3 ± 0.4 | 1.8 ± 0.2 |
| ***∆morA*** | 1.4 ± 0.3 | 2.9 ± 0.8 | 1.8 ± 0.5 | 1.6 ± 0.1 | 2.3 ± 0.1 | 1.9 ± 0.2 |
| ***∆spuC*** | 1.5 ± 0.3 | 2.0 ± 0.6 | 2.1 ± 0.3 | **1.9 ± 0.3*** | 2.3 ± 0.4 | 1.8 ± 0.1 |
| ***∆uvrA*** | 1.5 ± 0.1 | 2.1 ± 0.1 | 2.1 ± 0.5 | 1.6 ± 0.2 | 2.4 ± 0.4 | 2.0 ± 0.1 |
| ***∆puuA*** | 1.2 ± 0.2 | 2.5 ± 0.4 | 2.1 ± 0.3 | 1.3 ± 0.3 | 2.6 ± 0.6 | 1.7 ± 0.2 |
| ***∆katB*** | 1.6 ± 0.3 | 1.9 ± 0.4 | 2.4 ± 0.4 | **2.1 ± 0.5*** | 2.1 ± 0.1 | 1.9 ± 0.4 |
