# Supplementary material for: A Genome-Wide Screen Identifies Genes in Rhizosphere-Associated Pseudomonas Required to Evade Plant Defenses
Source: mBio. 2018 Nov 6;9(6):e00433-18. doi: 10.1128/mBio.00433-18 (PMC6222131; doi:10.1128/mBio.00433-18)
Supplement: TABLE S2 [file mbo005184154st2.docx]

|  | **Rhizosphere** | **LB** | **M9 succinate** | **Root Exudate** |
| --- | --- | --- | --- | --- |
| WCS365 | 0.50 ± 0.02 | 0.52 ± 0.03 | 0.49 ± 0.02 | 0.51 ± 0.02 |
| *∆colR* | **0.17 ± 0.11**** | **0.27 ± 0.05*** | **0.38 ± 0.04*** | **0.40 ± 0.04*** |
| *∆wapA* | **0.21 ± 0.02**** | 0.47 ± 0.01 | 0.46 **±** 0.01 | **0.44 ± 0.03*** |
| *∆cioA* | **0.37 ± 0.06*** | 0.52 ± 0.01 | 0.48 ± 0.01 | **0.37 ± 0.03*** |
| *∆gtsB* | **0.15 ± 0.08**** | **0.13 ± 0.06**** | **0.38 ± 0.03*** | **0.41 ± 0.02*** |
| *∆morA* | **0.35 ± 0.05**** | 0.54 ± 0.04 | 0.47 ± 0.03 | 0.44 ± 0.10 |
| *∆spuC* | **0.20 ± 0.05**** | **0.28 ± 0.02**** | **0.38 ± 0.02*** | 0.47 ± 0.05 |
| *∆uvrA* | **0.17 ± 0.07**** | 0.46 ± 0.04 | 0.50 ± 0.11 | 0.51 ± 0.02 |
| *∆puuA* | **0.40 ± 0.05*** | 0.41 ± 0.04 | 0.35 ± 0.17 | 0.44 ± 0.10 |
| *∆katB* | **0.26 ± 0.02**** | **0.33 ± 0.02*** | 0.43 ± 0.03 | 0.48 ± 0.07 |
