# Supplementary material for: A Genome-Wide Screen Identifies Genes in Rhizosphere-Associated Pseudomonas Required to Evade Plant Defenses
Source: mBio. 2018 Nov 6;9(6):e00433-18. doi: 10.1128/mBio.00433-18 (PMC6222131; doi:10.1128/mBio.00433-18)

A

STEP 1: DIGEST W/ MmeI

```
5'    5PHOS NNNNNNNNNNNNNNNNACAGGTTGGA TRANSPOSON TCCAACCTGTNNNNNNNNNNNNNNNNN      3'
3'        NNNNNNNNNNNNNNNNNNTGTCCAACCT NOSOPSNART AGGTTGGACTNNNNNNNNNNNNNNN  5PHOS 5'
```

STEP 2: PHOSPHOTASE DNA

```
5'          NNNNNNNNNNNNNNNNACAGGTTGGA TRANSPOSON TCCAACCTGTNNNNNNNNNNNNNNNNN      3'
3'        NNNNNNNNNNNNNNNNNNTGTCCAACCT NOSOPSNART AGGTTGGACTNNNNNNNNNNNNNNN        5'
```

STEP 3: LIGATE ADAPTOR

Adapter:

```
5'        ACACTCTTTCCCTACACGACGCTCTTCCGATCTNN
3' 3Phos  TGTGAGAAAGGGATGTGCTGCGAGAAGGCTAGA    5Phos
```

Final Product:

```
5' ACACTCTTTCCCTACACGACGCTCTTCCGATCTNN NNNNNNNNNNNNNNNACAGGTTGGA TRANSPOSON AGGTTGGACTNNNNNNNNNNNNNNNN AGATCGGAAGAGCGTCGTGTAGGGAAAGAGTGT 3'
3' TGTGAGAAAGGGATGTGCTGCGAGAAGGCTAGA NNNNNNNNNNNNNNNNNTGTCCAACCT NOSOPSNART TCCAACCTGTNNNNNNNNNNNNNN NNTCTAGCCTTCTCGCAGCACATCCCTTTCTCACA 5'
```

STEP 4: PCR AMPLIFY TRANSPOSON ENDS

```
PCR.1  5'  CAAGCAGAAGACGGCATACGAGATCGTGATGTGACTGGAGTTCAGACGTGTGCTCTTCCGATCTAGACCGGGGACTTATCATCCAACCTGT   3'
U.     5'  AATGATACGGCGACCACCGAGATCTACACTCTTTCCCTACACGACGCTCTTCCGATCT                                   3'
```

P7 FLOW CELL BINDING   BARCODE   INDEXING PRIMING SITE   TRANSPOSON   GENOMIC DNA   PRIMER READ 1 SITE   P5 FLOW CELL BINDING

```
5' CAAGCAGAAGACGGCATACGAGATCGTGATGTGACTGGAGTTCAGACGTGTGCTCTTCCGATCTAGACCGGGGACTTATCATCCAACCTGT
                                                                   TRANSPOSON TCCAACCTGTNNNNNNNNNNNNNNNNN AGATCGGAAGAGCGTCGTGTAGGGAAAGAGTGT
                                                                   NOSOPSNART AGGTTGGACTNNNNNNNNNNNNNNN NNTCTAGCCTTCTCGCAGCACATCCCTTTCTCACA
                                                                                                          TCTAGCCTTCTCGCAGCACATCCCTTTCTCACATCTAGAGCCACCAGCGGCATAGTAA 3'
```

Final Product:

P7 FLOW CELL BINDING   BARCODE   INDEXING PRIMING SITE   TRANSPOSON (MmeI site)   GENOMIC DNA   PRIMER READ 1 SITE   P5 FLOW CELL BINDING

```
5' CAAGCAGAAGACGGCATACGAGAT CGTGAT GTGACTGGAGTTCAGACGTGTGCTCTTCCGATCT AGACCGGGGACTTATCATCCAACCTGT NNNNNNNNNNNNNNNN AGATCGGAAGAGCGTCGTGTAGGGAAAGAGTGT AGATCTCGGTGGTCGCCGTATCATT 3'
```

B

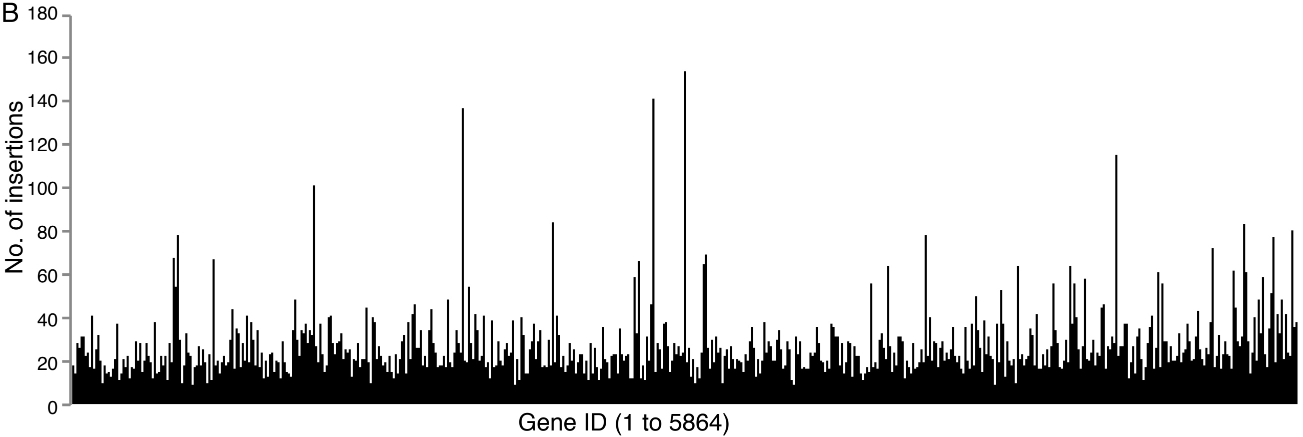

Supplement: FIG S3 [file mbo005184154sf3.pdf]
